# Unique H_2_-utilizing lithotrophy in serpentinite-hosted systems

**DOI:** 10.1038/s41396-022-01197-9

**Published:** 2022-10-07

**Authors:** Masaru Konishi Nobu, Ryosuke Nakai, Satoshi Tamazawa, Hiroshi Mori, Atsushi Toyoda, Akira Ijiri, Shino Suzuki, Ken Kurokawa, Yoichi Kamagata, Hideyuki Tamaki

**Affiliations:** 1 Bioproduction Research Institute, National Institute of Advanced Industrial Science and Technology (AIST), 1-1-1 Higashi, Ibaraki, Tsukuba, 305-8566 Japan; 2 Bioproduction Research Institute, National Institute of Advanced Industrial Science and Technology (AIST), 2-17-2-1, Tsukisamu-Higashi, Sapporo, 062-8517 Japan; 3 Horonobe Research Institute for the Subsurface Environment (H-RISE), Northern Advancement Center for Science & Technology, 5-3 Sakaemachi, Horonobe, Teshio, Hokkaido 098-3221 Japan; 4 National Institute of Genetics, 1111 Yata, Mishima, Shizuoka 411-8540 Japan; 5 Kochi Institute for Core Sample Research, Japan Agency for Marine-Earth Science and Technology (JAMSTEC), 200 Monobe Otsu, Nankoku, Kochi Japan; 6 Institute for Extra-Cutting-Edge Science and Technology Avant-garde Research (X-star), JAMSTEC, Natsushima 2-15, Yokosuka, Kanagawa 237-0061 Japan; 7 Institute of Space and Astronautical Science (ISAS), Japan Aerospace Exploration Agency (JAXA), 3-1-1 Yoshinodai, Chuo-ku, Sagamihara, Kanagawa 252-5210 Japan

**Keywords:** Environmental microbiology, Microbial ecology

## Abstract

Serpentinization of ultramafic rocks provides molecular hydrogen (H_2_) that can support lithotrophic metabolism of microorganisms, but also poses extremely challenging conditions, including hyperalkalinity and limited electron acceptor availability. Investigation of two serpentinization-active systems reveals that conventional H_2_-/CO_2_-dependent homoacetogenesis is thermodynamically unfavorable in situ due to picomolar CO_2_ levels. Through metagenomics and thermodynamics, we discover unique taxa capable of metabolism adapted to the habitat. This included a novel deep-branching phylum, “*Ca*. Lithacetigenota”, that exclusively inhabits serpentinite-hosted systems and harbors genes encoding alternative modes of H_2_-utilizing lithotrophy. Rather than CO_2_, these putative metabolisms utilize reduced carbon compounds detected in situ presumably serpentinization-derived: formate and glycine. The former employs a partial homoacetogenesis pathway and the latter a distinct pathway mediated by a rare selenoprotein—the glycine reductase. A survey of microbiomes shows that glycine reductases are diverse and nearly ubiquitous in serpentinite-hosted environments. “*Ca*. Lithacetigenota” glycine reductases represent a basal lineage, suggesting that catabolic glycine reduction is an ancient bacterial innovation by Terrabacteria for gaining energy from geogenic H_2_ even under hyperalkaline, CO_2_-poor conditions. Unique non-CO_2_-reducing metabolisms presented here shed light on potential strategies that extremophiles may employ for overcoming a crucial obstacle in serpentinization-associated environments, features potentially relevant to primordial lithotrophy in early Earth.

## Introduction

Serpentinite-hosted systems are rare and extreme habitats in which a hydrothermal process, serpentinization, alters ultramafic mantle rocks and yields hyperalkaline fluid rich in molecular hydrogen (H_2_) and reduced one-carbon compounds [1–8]. These fluids are often electron acceptor depleted—oxygen, nitrate, sulfate, etc. are absent (i.e., anoxic) and even the least favorable exogenous acceptor, carbon dioxide (CO_2_), is limiting due to the high alkalinity. Though previous studies explore the diversity of organisms in serpentinite-hosted systems, we have little insight into how indigenous H_2_-utilizing microorganisms combat the unique metabolic challenges in situ. One recent study shows strategies that methane-generating archaea employ to oxidize H_2_ in situ [9], but how other microorganisms (i.e., H_2_-utilizing anaerobic bacteria) overcome the electron acceptor limitation is poorly understood. Further, given that life is theorized to have emerged as H_2_-utilizing lithotrophs in early Earth serpentinite-hosted systems [10–24], modern lithotrophs inhabiting such ecosystems may represent valuable extant windows into the metabolism of primordial organisms. In this study, we pair metagenomics and thermodynamics to characterize uncultured putative anaerobic H_2_ utilizers inhabiting alkaline H_2_-rich serpentinite-hosted systems (Hakuba Happo hot springs in Hakuba, Japan, and The Cedars springs in California, USA; pH ~10.9 and ~11.9, respectively [25–27]) and elucidate novel, potentially ancient, lithotrophic strategies.

### Thermodynamics and geochemistry

The two primary strategies for utilizing H_2_ under anoxic conditions without favorable exogenous electron acceptors are methanogenesis and homoacetogenesis. As bacteria were detected in both Hakuba and The Cedars, yet archaea were absent in Hakuba, we focused our analyzes on metabolic strategies supporting bacterial H_2_ utilization (i.e., homoacetogenesis). To evaluate whether homoacetogenesis is viable in situ, we examined the in situ geochemical environment and the thermodynamics of H_2_/formate utilization and homoacetogenesis. The spring waters of both Hakuba and The Cedars contained H_2_ (e.g., 201–664 μM in Hakuba [27]). Formate, another compound thought to be abiotically generated through serpentinization, was also detected in Hakuba (8 μM in drilling well #3 [28]) and The Cedars (6.9 µM in GPS1). Acetate has also been detected in situ (4 μM in Hakuba [28] and 69.3 µM in The Cedars GPS1), suggesting these ecosystems may host novel H_2_- and/or formate-utilizing homoacetogens. Thermodynamic calculations using newly measured and published geochemical data (Tables S1 and S2) confirmed that H_2_ and formate are reductants in situ (i.e., H_2_ = 2H^+^ + 2e^−^/Formate^−^ = H^+^ + CO_2_ + 2e^−^): the Gibbs free energy yields (∆*G*) for oxidation (coupled with physiological electron carriers NADP^+^, NAD^+^, and ferredoxin) are less than −4.78 kJ per mol H_2_ and −24.92 kJ per mol formate in Hakuba, and −10.73 and −22.03 in The Cedars respectively (H_2_ concentration was not available for The Cedars so the highest concentration observed in Hakuba [664 µM] was used; see Supplementary Results). However, serpentinite-hosted systems impose a unique challenge to homoacetogenesis—a key substrate, CO_2_, is at extremely low concentrations due to the high alkalinity. We estimate that the aqueous CO_2_ concentration is below 0.0006 nM in Hakuba (pH 10.7 and <0.1 µM TIC) and 0.003 nM in The Cedars (pH 11.9 and 35 µM TIC) (Table S2) [25, 27]. In Hakuba, H_2_/CO_2_-driven acetogenesis (∆*G* of −3.71 kJ per mol acetate) cannot support microbial energy generation (∆*G* ≤ −20 kJ per mol is necessary [29]; Fig. S1). Moreover, in both Hakuba and The Cedars, one of the first steps in CO_2_-reducing homoacetogenesis, reduction of CO_2_ to formate, is unfavorable based on the thermodynamics presented above (∆*G* > +24.92 or +22.02 kJ per mol formate). Thus, catabolic reduction of CO_2_ to acetate is thermodynamically challenging in situ and may only run if investing ATP (e.g., Calvin–Benson–Bassham cycle [−6 ATP; ∆*G* of −361.68 kJ per mol acetate in Hakuba] or reductive tricarboxylic acid [−1 ATP; −61.68 kJ per mol]). Under CO_2_ limitation, autotrophs are known to accelerate CO_2_ uptake through HCO_3_^−^ dehydration to CO_2_ (carbonic anhydrase) or carbonate mineral dissolution, but both only modify kinetics and are not effective in changing the maximum CO_2_ concentration (determined by equilibrium with carbonate species). In addition, in Hakuba, the CO_3_^2−^ concentration is too low (84.7 nM CO_3_^2−^) to cause carbonate mineral precipitation (e.g., [CO_3_^2−^] must exceed 38.5 µM given *K*_s_ of 5 ×  10^−9^ for CaCO_3_ and [Ca^2+^] of 0.13 mM).

Based on thermodynamic calculations, the energy obtainable from H_2_/CO_2_-driven homoacetogenesis is too small to support life in many serpentinite-hosted systems, yet acetate is detected in some of these ecosystems (Fig. S2 and Table S2; note that we cannot exclude the possibility that acetate may be produced abiotically by water–rock reactions [30]). Thus, CO_2_-independent electron-disposing metabolism may have been necessary for extremophilic organisms to gain energy from H_2_ in the hyperalkaline fluids of hydrothermal systems. Here, we explore the metabolic capacities of organisms living in serpentinite-hosted systems to gain insight into potential metabolic strategies for utilizing H_2_ under the extreme conditions in situ.

### Diverse putative H_2_- and formate-utilizing organisms

**Fig. 1:**
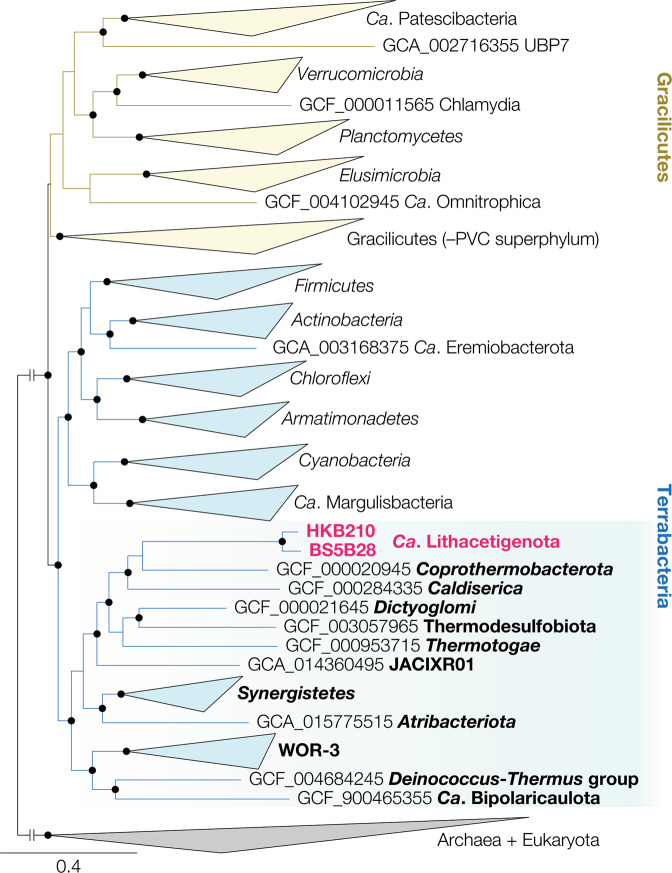
Ribosomal protein tree including high-quality MAGs from 74 GTDB-defined phylum-level lineages. Representative genomes (highest quality based on a score defined as completeness – 5*contamination, both estimated by CheckM) were chosen for bacterial classes that contain at least one genome the meet the following criteria: (i) cultured organisms with ≥90% completeness, ≤5% contamination (as estimated by CheckM), and ≤ 20 contigs; (ii) uncultured organisms with ≥85% completeness, ≤3% contamination, and ≤20 contigs; and (iii) *Ca.* Patescibacteria with ≥60% completeness and ≤1 contig. Universally conserved ribosomal proteins were collected from each genome, aligned with MAFFT v7.394, trimmed with BMGE v1.12 (-m BLOSUM30 -g 0.67 -b 3), and concatenated. A maximum likelihood tree was calculated using IQ-TREE v2.1.3 with the UDM0064LCLR model (-m Poisson+UDM0064LCLR), ultrafast bootstrap approximation, and SH-like approximate likelihood ratio test (-B 1000 -alrt 1000; bootstrap values are recalculated with BOOSTER using the -tbe option). Branches with ≥90% ultrafast bootstrap support and ≥80% SH-alrt support are indicated with black circles. Archaeal and eukaryotic genomes were used as an outgroup. The inter-domain branch was shortened with a break to 1/10 of the calculated length for illustrative purposes. Phylogenetic groups corresponding to “Gracilicutes” and “Terrabacteria” are indicated yellow and blue respectively. *Ca.* Lithacetigenota are highlighted (magenta). See Supplementary Fig. S4 for full tree.

**Fig. 2:**
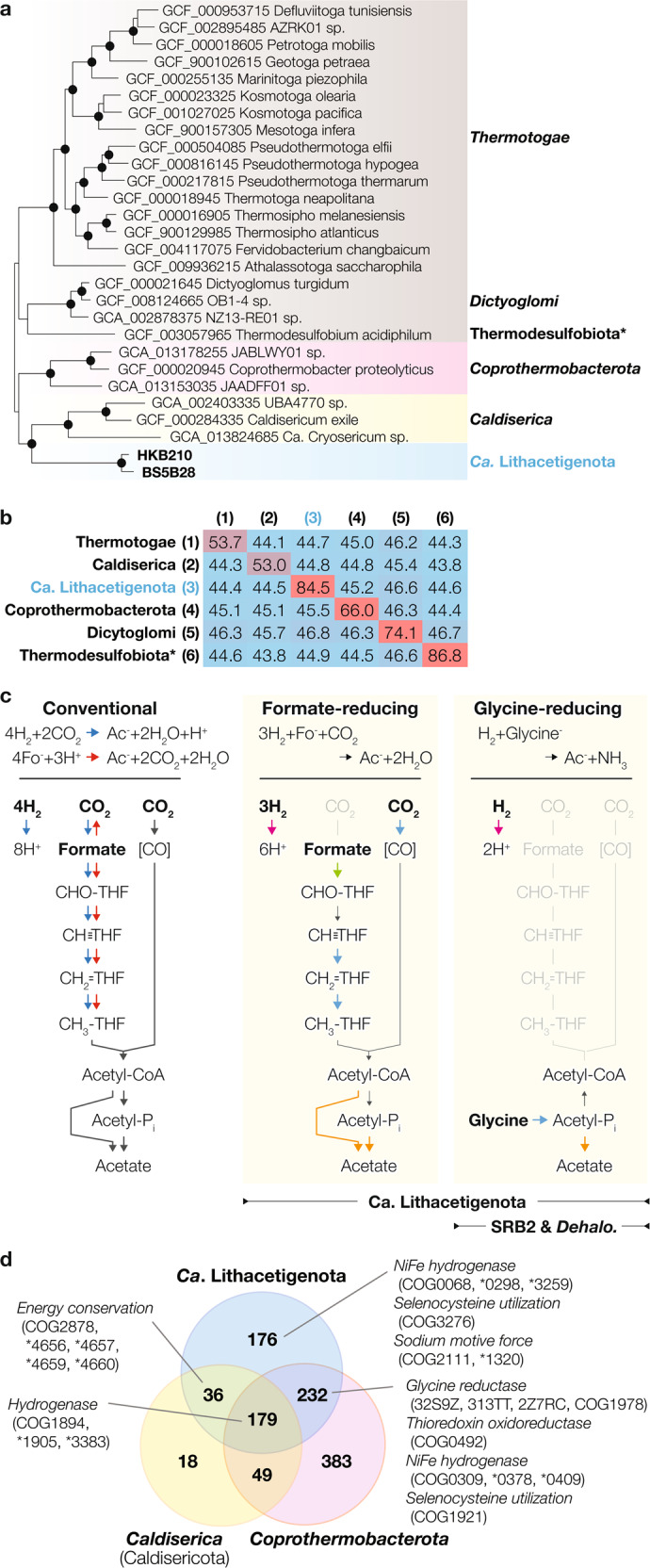
“*Ca*. Lithacetigenota” phylogeny, lithotrophic acetate generation pathways, and comparative genomics with neighboring phyla. **a** A maximum likelihood tree was calculated for a concatenated alignment of universally conserved ribosomal protein sequences from representative genomes of individual phyla (aligned with MAFFT v7.394 [default parameters] and trimmed with BMGE v1.12 (−m BLOSUM30 −g 0.67 −b 3) using IQ-TREE v2.1.3 with the UDM0064LCLR model (-m Poisson+UDM0064LCLR), ultrafast bootstrap approximation, and SH-like approximate likelihood ratio test (-B 1000 -alrt 1000; bootstrap values are recalculated with BOOSTER using the -tbe option). Branches with ≥90% ultrafast bootstrap support and ≥80% SH-alrt support are indicated with black circles. Phylum names are shown for NCBI taxonomy (italicized) or GTDB classification (*). **b** The average inter-phylum AAI (as calculated by CompareM) was calculated using GTDB species representatives. **c** Putative metabolic pathways potentially adapted to the CO_2_-limited hyperalkaline conditions encoded by “*Ca*. Lithacetigenota” members and others: formate- and glycine-reducing acetate generation. Arrow colors indicate oxidative (pink), reductive (blue), ATP-yielding (orange), and ATP-consuming (green) steps. **d** Venn diagram of COGs/NOGs (as predicted by eggnog-mapper) fully conserved across all members of each phylum (genomes included in GTDB release 95 with completeness ≥85% and contamination ≤5%). COGs/NOGs related to lithotrophy and alkaliphily are highlighted. * “COG” abbreviated.

Through metagenomic exploration of the two serpentinite-hosted systems (Table S3), we discover a plethora of phylogenetically novel organisms encoding genes for H_2_ and formate metabolism (19 bins with 73.2–94.8% completeness and 0.0–8.1% contamination [86.1% and 3.8% on average respectively]; available under NCBI BioProject PRJNA453100) despite challenges in acquisition of genomic DNA (15.7 and 18.9 ng of DNA from 233 and 720 L of filtered Hakuba Happo spring water, respectively; RNA was below the detection limit). We find metagenome-assembled genomes (MAGs) affiliated with lineages of *Firmicutes* (e.g., *Syntrophomonadaceae* and uncultured family SRB2), *Actinobacteria*, and candidate division NPL-UPA2 [31] (Fig. S3). We also recovered MAGs for a novel lineage, herein referred to as “*Ca*. Lithacetigenota”, that inhabits both Hakuba and The Cedars and, to our knowledge, no other ecosystems (Figs. 1, 2a, S3, and S4). The average amino acid identity (AAI) between *Ca*. Lithacetigenota and neighboring phyla (*Coprothermobacterota*, *Dictyoglomi*, Thermodesulfobiota [GTDB-defined phylum], *Thermotogae*, and *Caldiserica*) was comparable to the average interphylum AAI among the neighboring phyla (45.33 ± 0.86% vs 45.17 ± 0.99%), suggesting that *Ca*. Lithacetigenota represents a novel phylum-level lineage (Fig. 2a, b). These genomes encode enzymes for oxidizing H_2_ and formate (i.e., hydrogenases and formate dehydrogenases [32–39]; see Supplementary Results), suggesting that organisms in situ can employ H_2_ and formate as electron donors.

### “*Ca*. Lithacetigenota” has unique site-adapted metabolism

Inspection of the serpentinite-hosted environment-exclusive phylum “*Ca*. Lithacetigenota” reveals specialization to H_2_-driven lithotrophy potentially suitable for the low-CO_2_ in situ conditions (Fig. 2c). We discover that The Cedars-inhabiting population (e.g., MAG BS5B28, 94.8% completeness and 2.9% contamination) harbors genes for H_2_ oxidation ([NiFe] hydrogenase Hox), a nearly complete Wood-Ljungdahl pathway, and an oxidoreductase often associated with acetogenesis—NADH:ferredoxin oxidoreductase Rnf [40, 41] (Tables S4 and S5). One critical enzyme, the formate dehydrogenase, is missing from all three “*Ca*. Lithacetigenota” MAGs from The Cedars (and unbinned contigs), indicating that these bacteria can neither perform H_2_/CO_2_-driven nor formate-oxidizing acetogenesis (Fig. 2c). However, even without the formate dehydrogenase, the genes present can form a coherent pathway that uses formate rather than CO_2_ as a starting point for the “methyl branch” of the Wood–Ljungdahl pathway (i.e., formate serves as an electron acceptor; Fig. 2c). This is a simple yet potentially effective strategy for performing homoacetogenesis while circumventing the unfavorable reduction of CO_2_ to formate. Coupling H_2_ oxidation with this formate-reducing pathway is thermodynamically viable as it halves the usage of CO_2_ (3H_2_ + Formate^−^ + CO_2_ = Acetate^−^ + 2H_2_O; ∆*G* of −29.62 kJ per mol acetate) and, as a pathway, is simply an intersection between the conventional H_2_/CO_2_-driven and formate-disproportionating acetogenesis (Fig. 2c and S5). Although use of formate as an electron acceptor for formate-oxidizing acetogenesis is quite common, no previous homoacetogens have been observed to couple H_2_ oxidation with acetogenesis from formate, likely because CO_2_ has a much higher availability than formate in most ecosystems.

The Hakuba-inhabiting “*Ca*. Lithacetigenota” (HKB210 and HKB111) also encodes Hox for H_2_ oxidation but lacks genes for homoacetogenesis (no homologs closely related to The Cedars population genes were detected even in unbinned metagenomic contigs). We suspect that this population forgoes the above H_2_/formate-driven homoacetogenesis because the estimated energy yield of the net reaction in situ (∆*G* of −19.94 kJ per mol acetate) is extremely close to the thermodynamic threshold of microbial catabolism (slightly above −20 kJ per mol) and, depending on the actual threshold for “*Ca*. Lithacetigenota” and/or even slight changes in the surrounding conditions (e.g., ∆*G* increases by 1 kJ per mol if H_2_ decreases by 20 µM decreases in Hakuba), the metabolism may be unable to recover energy. Through searching the physicochemical environment for alternative exogenous electron acceptors and MAGs for electron-disposing pathways, we detected a low concentration of glycine in situ (5.4 ± 1.6 nM; Table S6) and found genes specific to catabolic glycine reduction (see next paragraph). We suspect that some portion of this glycine is likely geochemically generated in situ, given that (a) glycine is often detected as the most abundant amino acid produced by both natural and laboratory-based serpentinization (e.g., H_2_ + Formate = Formaldehyde ⇒ Formaldehyde + NH_3_ = Glycine) [10, 16, 42–47] and (b) no other amino acid was consistently detectable (if glycine was cell-derived, other amino acids ought to also be consistently detected).

**Fig. 3:**
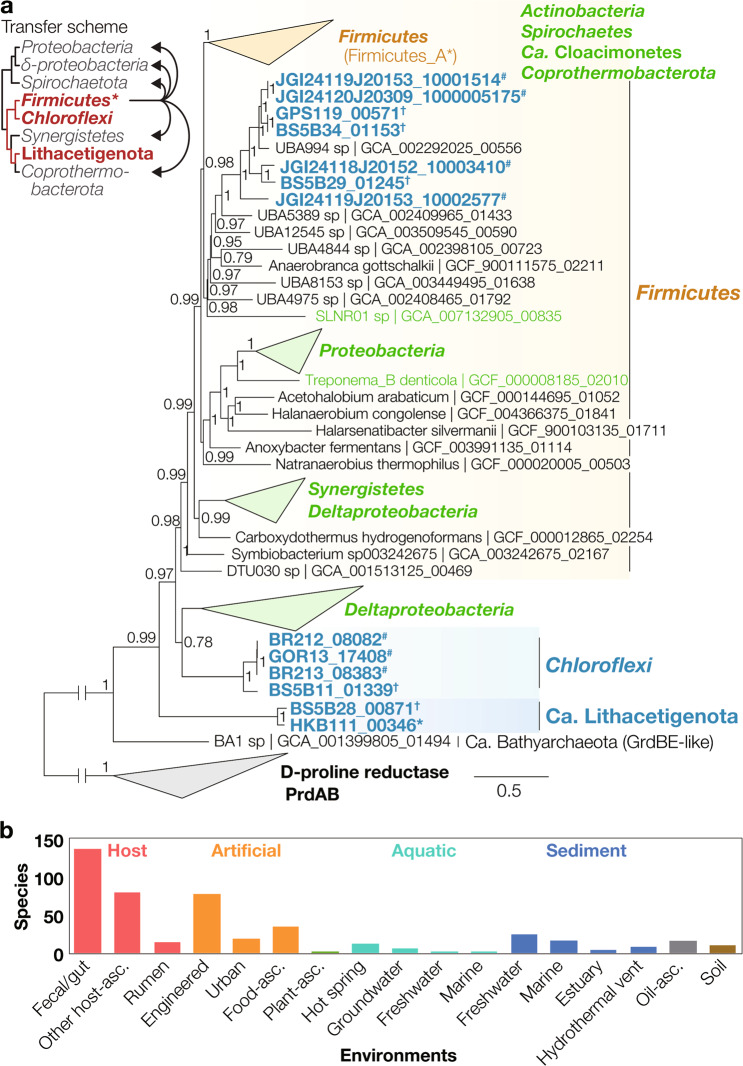
Evolution and distribution of glycine reductases. **a** Phylogeny of serpentinite-hosted microbiome glycine reductase subunit GrdBE homologs (Hakuba Happo hot spring*, The Cedars springs^†^, and other serpentinite-hosted system metagenomes^#^) and a brief scheme for evolutionary history of Grd. Grd-related COG1978 homologs were collected from the representative species genomes in GTDB, filtered using a GrdB motif conserved across members of phyla known to perform glycine-reducing Stickland reaction (see Methods and Supplementary Fig. S6) and clustered with 75% amino acid sequence similarity using CD-HIT (-c 0.75). GrdB-related sarcosine reductase subunits were excluded by identification of a GrdF motif conserved across sequences that form a distinct cluster around the biochemically characterized *Peptoclostridium acidaminophilum* GrdF. GrdE neighboring GrdB were collected. D-proline reductase subunits PrdBA (homologous to GrdB and GrdE respectively) was used as an outgroup. GrdB+PrdB and GrdE+PrdA were aligned (MAFFT v7.394) and trimmed (BMGE v1.12 -m BLOSUM30 -g 0.05) separately, then concatenated. A maximum likelihood tree was calculated using IQ-TREE v2.1.3 (-m LG+C20+G+F) and 1000 ultrafast bootstrap replicates (bootstrap values are recalculated with BOOSTER). Branches with ≥95% ultrafast bootstrap support are indicated with pink circles. Serpentinite-hosted system-derived sequences are shown in blue and taxa that may have gained GrdB through horizontal transfer are shown in green. Though the GrdB motif did not match, the closest (and only) detectable archaeal homolog (COG1978) identified in *Ca*. Bathyarchaeota is included. An axis break is used for the branch connecting GrdBE (and the *Ca*. Bathyarchaeota homolog) and outlier PrdBA for readability (10% of actual length). See Supplementary Fig. S6 for complete tree and full branch length between GrdBE and PrdBA. In the brief scheme of Grd evolution (top left), the cladogram topology is based on Fig. S4. Vertical transfer (red lines in cladogram) and horizontal transfer (black arrows) inferred from tree structures are shown. Phyla that may have acquired Grd vertically (red) and horizontally (gray) are indicated. GTDB phyla belonging to *Firmicutes* were grouped together. * GTDB-defined phylum-level lineage nomenclature. **b** Number of glycine reductase-encoding GTDB-defined species representatives (GTDB r95) associated with different environments. Only genomes with both GrdB and GrdE were included.

For utilization of the putatively abiotic glycine, the Hakuba “*Ca*. Lithacetigenota” encodes glycine reductases (Grd; Fig. 3 and S6; Tables S4 and S5)—a unidirectional selenoprotein for catabolic glycine reduction [48, 49]. Based on the genes available, this population likely specializes in coupling H_2_ oxidation and glycine reduction (H_2_ + Glycine^−^ → Acetate^−^ + NH_3_; Fig. 2c). Firstly, the genomes encode NADP-linked thioredoxin reductases (NADPH + Thioredoxin_ox_ → NADP^+^ + Thioredoxin_red_) that can bridge electron transfer from H_2_ oxidation (H_2_ + NADP → NADPH + H^+^) to glycine reduction (Glycine^−^ + Thioredoxin_red_ → Acetyl-_Pi_ + NH_3_ + Thioredoxin_ox_). Secondly, though glycine reduction is typically coupled with amino acid oxidation (*i.e*., Stickland reaction in *Firmicutes* and *Synergistetes* [48, 50]), similar metabolic couplings have been reported for some organisms (i.e., formate-oxidizing glycine reduction [via Grd] [51] and H_2_-oxidizing trimethylglycine reduction [via Grd-related betaine reductase] [52]). Thirdly, Grd is a rare catabolic enzyme, so far found in organisms that specialize in amino acid (or peptide) catabolism, many of which are reported to use glycine for the Stickland reaction (e.g., *Peptoclostridium* of *Firmicutes* and *Aminobacterium* of *Synergistetes* [53]). Lastly, the population lacks any discernable fermentative (propionate [methylmalonyl-CoA pathway], butyrate [reverse beta oxidation], lactate [lactate dehydrogenase], and alanine [alanine dehydrogenase]) and respiratory (aerobic [terminal oxidases], nitrate [nitrate reductase, nitrite reductase, nitric oxide reductase, nitrous oxide reductase], sulfate [dissimilatory sulfate reductase and sulfite reductase], other sulfurous compounds [molybdopterin-binding protein family sulfurous compound reductases], and metals [outer membrane cytochrome OmcB]) electron disposal pathways and oxidative organotrophy (Tables S4 and S5). Although the BS5B28 genome encodes a bifunctional alcohol/aldehyde dehydrogenase and aldehyde:ferredoxin oxidoreductase, no complete sugar or amino acid degradation pathways could be identified, suggesting that these genes have a physiological role unrelated to ethanol fermentation. Further, though formate and glycine transporters were absent in the genomes, a survey of transporters (annotated in UniProtKB 2021_03 [54]) revealed that no alkaliphiles (organisms with optimum pH ≥ 9.5 in the DSMZ BacDive database [55]) encoded known formate transporters (focA; TIGR04060) or amino acid permeases (PF00324) (ABC transporters were not considered as substrate specificity for these complexes cannot be annotated reliably), indicating that alkaliphiles likely employ unknown transport proteins. Reflecting the lack of other catabolic pathways, the Hakuba “*Ca*. Lithacetigenota” MAGs display extensive genome streamlining, comparable to that of *Aurantimicrobium* [56, 57], “*Ca*. Pelagibacter” [58], and *Rhodoluna* [59] in aquatic systems, as also reported for other organisms inhabiting serpentinite-hosted systems [60, 61] (Fig. S7). Thermodynamic calculations show that H_2_-oxidizing glycine reduction is favorable in situ (∆*G*°’ of −70.37 kJ per mol glycine [∆*G* of −85.84 in Hakuba]; Fig. S1). Further, based on the pathway identified, this putative metabolism is >10 times more efficient in recovering energy from H_2_ (1 mol ATP per mol H_2_) than acetogenesis utilizing H_2_/CO_2_ (0.075 mol ATP per mol H_2_ based on the pathway *Acetobacterium woodii* utilizes) or H_2_/formate (0.075 mol ATP per mol H_2_, assuming no energy recovery associated with the formate dehydrogenase). We also detect glycine reductases in The Cedars “*Ca*. Lithacetigenota”, indicating that it may also perform this metabolism (∆*G* of −76.87 in The Cedars, assuming 201 µM H_2_).

Given the phylogenetic and metabolic uniqueness of these populations, we report provisional taxonomic assignment to “*Ca*. Lithacetigenota” phyl. nov., “*Ca*. Lithacetigena glycinireducens” gen. nov., sp. nov. (HKB111 and HKB210), and “*Ca*. Psychracetigena formicireducens” gen. nov., sp. nov. (BS525, BS5B28, and GPS1B18) (see Supplementary Results). Based on a concatenated ribosomal protein tree, this serpentinite-hosted ecosystem-associated candidate phylum is closely related to the deepest-branching group of bacterial phyla in “Terrabacteria”, one of the two major of lineages *Bacteria* (Fig. 1). Comparative genomics shows that “*Ca*. Lithacetigenota” shares 623 core functions (based on Bacteria-level COGs/NOGs predicted by eggnog-mapper shared by the two highest quality Hakuba and The Cedars MAGs HKB210 and BS5B28; Fig. 2d). When compared with the core functions of two closest related phyla (*Caldiserica* and *Coprothermobacterota*), 176 functions were unique to “*Ca*. Lithacetigenota”, including those for NiFe hydrogenases (and their maturation proteins), selenocysteine utilization (essential for Grd), and sodium:proton antiporter for alkaliphily. With *Coprothermobacterota*, 232 functions were shared, including Grd, thioredoxin oxidoreductase (essential for electron transfer to Grd), and additional proteins for NiFe hydrogenases and selenocysteine utilization, pointing toward importance of H_2_ metabolism and glycine reduction for these closely related phyla. More importantly, among bacterial phyla in the deep-branching group, “*Ca*. Lithacetigenota” represents the first lineage inhabiting hyperalkaliphilic serpentinite-hosted ecosystems, suggesting that these organisms may be valuable extant windows into potential physiologies of primordial organisms who are thought to have lived under hyperalkaline conditions (albeit with 4 billion years of evolution in between; see discussion regarding Grd below).

### Widespread glycine reduction in serpentinite-hosted systems

Uncultured members of *Chloroflexi* (Chloroflexota) class *Dehalococcoidia* inhabiting The Cedars and *Firmicutes* (Firmicutes_D) class SRB2 in Hakuba and The Cedars also possess glycine reductases (Table S5). In addition, these populations encode hydrogenases and formate dehydrogenases, suggesting that they may also link H_2_ and formate metabolism to glycine reduction. Closely related glycine reductases were also detected in other studied serpentinite-hosted systems (47–94% amino acid similarity in Tablelands, Voltri Massif, and Coast Range Ophiolite) [1, 2, 7]. Phylogenetic analysis of the glycine-binding “protein B” subunits GrdB and GrdE reveals close evolutionary relationships between glycine reductases from distant/remote sites (Fig. 3a and S6). Note that Tablelands spring glycine reductase sequences were not included in the analysis as they were only detected in the unassembled metagenomic reads (4460690.3; 69.7–82.2% similarity to Hakuba SRB2). Overall, “*Ca*. Lithacetigenota”, *Dehalococcoidia*, and SRB2 glycine reductases are all detected in at least two out of the seven metagenomically investigated systems despite the diverse environmental conditions (e.g., temperature). Thus, we propose glycine as an overlooked thermodynamically and energetically favorable electron acceptor for H_2_ oxidation in serpentinite-hosted systems. We suspect that glycine reduction may be a valuable catabolic strategy as the pathway requires few genes/proteins (a hydrogenase, Grd, acetate kinase, and thioredoxin oxidoreductase) and conveniently provides acetate, ammonia, and ATP as basic forms of carbon, nitrogen, and energy.

Phylogenetic analysis of glycine reductases (Fig. 3a and S6) shows that the novel homologs recovered from serpentinite-hosted systems represent deep-branching lineages distantly related from those detectable in published genomes (GTDB r95 species representatives). Further comparison of the topology with a ribosomal protein-based genome tree (Fig. 2a) indicates that the two deep-branching serpentinite-hosted system-affiliated lineages (*Ca*. Lithacetigenota and novel *Chloroflexi* family) and *Firmicutes* vertically inherited glycine reductases. Thus, catabolic glycine reduction can be traced back to the concestor of these three lineages, suggesting the metabolism at least dates back to the ancestor of “Terrabacteria”. We further identified an archaeal GRD homolog (in Miscellaneous Crenarchaeota Group [MCG] or *Ca*. Bathyarchaeota member BA-1; Fig. 3a and S6), but whether this gene functions as a glycine reductase (GrdB motif not fully conserved) and, further, truly belong to this clade (source is metagenome-assembled genome) remains to be verified. Reconstruction of the ancestral Grd sequence and estimation of its pH preference (via AcalPred) showed that the ancestral enzyme likely had good efficiency under alkaline conditions (pH > 9; probability of 0.9973 and 0.9858 for GrdB and GrdE, respectively). Thus, the currently available data suggest that Grd (and catabolic glycine reduction) is an ancient bacterial catabolic innovation in an alkaline habitat, dating back to one of the deepest nodes in the bacterial tree.

While we detect glycine reductases in many serpentinite-hosted systems, examination of genomes derived from other natural ecosystems shows that only 107 species (species representatives in GTDB r95; 0.35% of all GTDB species) inhabiting such habitats encode GrdBE (Fig. 3b and S6). This is a level comparable to rare artificial contaminant-degrading enzymes (*e.g*., tetrachloroethane dehalogenase pceA—65 species [encoding KEGG KO K21647 based on AnnoTree with GTDB r95 and default settings [62]]; dibenzofuran dioxygenase—258 species [K14599 and K14600]). Most glycine reductase homologs are found in species affiliated with host-associated (mostly human body and rumen) or artificial habitats (360 species), the majority of which belong to the phylum *Firmicutes*. We suspect that glycine reduction has low utility in most natural ecosystems (e.g., no excess glycine via abiotic generation and no severe nutrient/electron acceptor limitation) and has been repurposed by some anaerobes for the fermentative Stickland reaction in organic-rich ecosystems (e.g., host-associated ecosystems) where excess amino acids are available but access to favorable electron acceptors is limited (Fig. 3b) (notably, glycine is the dominant amino acid in collagen [>30%], the most abundant protein in vertebrate bodies).

### Other characteristics of putative indigenous homoacetogens

In contrast with members of “*Ca*. Lithacetigenota”, several other putative homoacetogenic populations encode the complete Wood–Ljundgahl pathway (Tables S4 and S5), indicating that other forms of acetogenesis may also be viable in situ. One putative homoacetogen in The Cedars, NPL-UPA2, lacks hydrogenases but encodes formate dehydrogenases. Although the NPL-UPA2 population cannot perform H_2_/formate-driven acetogenesis, it may couple formate oxidation with formate-reducing acetogenesis—another thermodynamically viable metabolism (∆*G* of –50.90 kJ per mol acetate in The Cedars; Fig. S5). The pathway uses CO_2_ as a substrate but has lower CO_2_ consumption compared to H_2_/CO_2_ homoacetogenesis and can produce intracellular CO_2_ from formate. A recent study also points out that methanogens inhabiting serpentinite-hosted environments oxidize formate presumably to generate intracellular CO_2_ [9]. In Hakuba, an *Actinobacteria* population affiliated with the uncultured class UBA1414 (MAG HKB206) encodes hydrogenases and a complete Wood–Ljungdahl pathway (Table S5) and, thus, may be capable of H_2_/formate or the above formate-disproportionating acetogenesis (Fig. 2c and S5). Indeed, the UBA1414 population was enriched in Hakuba-derived cultures aiming to enrich acetogens using the H_2_ generated by the metallic iron–water reaction [63] (Fig. S8). Many populations encoding a complete Wood–Ljungdahl pathway possess monomeric CO dehydrogenases (CooS unassociated with CODH/ACS subunits; NPL-UPA2, *Actinobacteria*, *Syntrophomonadaceae* [Hakuba and The Cedars], and *Dehalococcoidia* [The Cedars]; Table S4). Although CO is below the detection limit in Hakuba (personal communication with permission from Dr. Konomi Suda), another study shows that CO metabolism takes place in an actively serpentinizing system with no detectable CO [64]. Given that CO is a known product of serpentinization [7, 64], it may be an important substrate for thermodynamically favorable acetogenesis in situ. However, further investigation is necessary to verify this (e.g., need to measure CO at multiple time points).

Another interesting adaptation observed for all putative homoacetogens detected in Hakuba and The Cedars was possession of an unusual CODH/ACS complex. Although *Bacteria* and *Archaea* are known to encode structurally distinct forms of CODH/ACS (designated as Acs and Cdh respectively for this study), all studied Hakuba/The Cedars putative homoacetogens encode genes for a hybrid CODH/ACS that integrate archaeal subunits for the CO dehydrogenase (AcsA replaced with CdhAB) and acetyl-CoA synthase (AcsB replaced with CdhC) and bacterial subunits for the corrinoid protein and methyltransferase components (AcsCDE) (Table S4). The *Firmicutes* lineages also additionally encode the conventional bacterial AcsABCDE. Given that all of the identified putative homoacetogens encode this peculiar hybrid complex, we suspect that such CODH/ACS’s may have features adapted to the high-pH low-CO_2_ conditions (e.g., high affinity for CO_2_ and/or CO). In agreement, a similar hybrid CODH/ACS has also been found in the recently isolated “*Ca*. Desulforudis audaxviator” inhabiting an alkaline (pH 9.3) deep subsurface environment with a low CO_2_ concentration (below detection limit [65, 66]) [67].

### Implications for primordial biology

The last universal common ancestor (LUCA) is hypothesized to have evolved within alkaline hydrothermal mineral deposits at the interface of serpentinization-derived fluid and ambient water (e.g., Hadean weakly acidic seawater) [22–24]. Although such interfaces no longer exist (i.e., ancient Earth lacked O_2_ but most water bodies contain O_2_ on modern Earth), modern anoxic terrestrial and oceanic ecosystems harboring active serpentinization [1–8] may hold hints for how primordial organisms utilized H_2_ under hyperalkaline CO_2_-depleted conditions (e.g., post-LUCA H_2_-utilizing organisms that ventured away from the interface towards the alkaline fluids). Our findings suggest that unconventional modes of lithotrophy that take advantage of geogenic reduced carbon compounds (e.g., formate and glycine) as exogenous electron acceptors may have been viable approaches to circumventing thermodynamic issues and obtaining energy from H_2_ oxidation in situ. The strategies we discover are largely exclusive to the bacterial domain (archaeal CO_2_ reduction does not involve formate as an intermediate and, to our knowledge, glycine reduction is limited to *Bacteria*) and originated deep in the bacterial tree, suggesting they may have been relevant in the divergence towards the bacterial and archaeal domains. Notably, the estimated alkaliphily of the ancestral Grd also points towards the relevance of this metabolism in ancient alkaline habitats.

## Conclusion

Through the investigation of serpentinite-hosted systems, we discover a novel habitat-exclusive alkaliphilic phylum that belongs to a deep-branching group of bacterial phyla and likely relies on putative site-adapted H_2_-oxidizing lithotrophy (e.g., coupled with formate or glycine reduction) thermodynamically favorable in the electron acceptor- and CO_2_-depleted conditions in situ. The consistent presence of catabolic glycine reductases across these habitats also indicates that glycine may be an important electron acceptor, potentially abiotically generated via serpentinization. Moreover, the identified glycine reductases represent hitherto overlooked deep-branching lineages that point toward antiquity of catabolic and potentially alkaline glycine reduction, suggesting thermodynamic and phylogenetic relevance to ancient metabolism in serpentinite-hosted systems. Further investigation of microbiology in these habitats may reveal novel organisms, metabolic strategies, and rare enzymes adapted to polyextreme conditions in Earth’s modern and ancient subsurface environments.

## Methods

### Sampling site and sample collection

The Hakuba Happo samples for geochemical and microbiological analysis were artificially pumped from a drilling well (700 m in depth), which was previously described and named Happo #3 (36°42′N 137°48′E [27]). For microbiological analysis, two spring water samples were taken at different time points, 233 L taken in July 2016 (labeled HKB701) and 720 L taken in October 2016 (labeled HKB702), respectively. To collect microbial cells, samples were filtered through a 0.1-μm Omnipore membrane filter (Merck Millipore) using a 90 mm diameter stainless-steel filter holder (Merck Millipore) attached to FDA Viton tubing (Masterflex) at a sampling site. After filtration, filters were immediately transferred to sterile tubes and frozen in a dry ice-ethanol bath, transported in dry ice, and stored at −80 °C until DNA extraction. Only in October 2017, water samples for NH_3_ and amino acid analysis were collected from the same well Happo #3, transferred to dry-heat-sterilized nitrogen-purged 100 ml glass vials, and stored at 4 °C.

### Geochemical analysis

The water temperature of hot spring water was measured using a thermometer (CT-430WP, Custom Ltd.) at a site. The pH, oxidation reduction potential (ORP), electrical conductivity (EC), and dissolved oxygen (DO) level were determined with portable devices, including a pH meter (D-23, Horiba), an ORP meter (RM-30P, TOA-DKK), an EC meter (CM-31P, TOA-DKK), and a DO meter (DO-31P, TOA-DKK), correspondingly (note that the water sample was pumped up from underground and immediately used for these measurements before cooling). The ion concentrations of Na^+^, K^+^, Ca^2+^, and NO_3_^-^ were determined using portable sensors (LAQUAtwin series, Horiba). The in situ NH_3_ concentration was determined by measuring aqueous NH_4_^+^ and gaseous NH_3_ (purged with N_2_ gas, gas dissolved into deionized water, and measured dissolved NH_4_^+^) of a sample stored as described above using high-performance liquid chromatography (HPLC; Prominence; Shimadzu), then adding the two together. For amino acid quantification, the sample was concentrated under a stream of nitrogen gas and then analyzed following Shimadzu protocol no. L323 (https://www.an.shimadzu.co.jp/hplc/prominence/l323.pdf) using HPLC with minor modifications (fluorescence detector RF-20Axs; sodium hypochlorite solution was not added for detection of proline). The Cedars spring concentrations of formate and acetate were determined by Isotope-Ratio-Monitoring Liquid Chromatography Mass Spectrometry; Thermo- Finnigan Delta Plus XP isotope-ratio mass spectrometer connected to LC IsoLink, as described by Heuer et al. [68] and Ijiri et al. [69].

### Thermodynamic calculations

The Hakuba and Cedars calculations Gibbs free energy yield (∆*G*) are based on ∆*G*°_*f*_ and ∆*H*°_*f*_ values at 298 K, respective pH (10.7 and 11.9), and adjustment to the in situ temperatures (48 and 17 °C) through the Gibbs–Helmholtz equation [70]. The effect of pressure was approximated as described by Wang et al. [71]. For both Hakuba and The Cedars calculations, the glycine concentration (5.4 nM) was based on measurements from Hakuba. Formate, acetate, and NH_3_ concentrations were based on respective measurements from Hakuba (8 µM formate, 4 µM acetate, and 2.9 µM NH_3_) and The Cedars (6.9 µM formate [average of 6.777 and 7.079 µM measured on September 2017], 69.3 µM acetate [average of 69.601 and 68.967 µM measured on September 2017], and 1 µM NH_3_ [below detection limit]). For Hakuba, the H_2_ concentration measured in Hakuba drilling well #3 (DNA source) was used (201 µM H_2_). For The Cedars, the highest detected H_2_ concentration in Hakuba was used (664 µM H_2_ in drilling well #1). See also Tables S1 and S2 and Supplementary Equations.

### Metagenome sequencing, assembly, and binning

The filter was aseptically cut into 16 equal pieces using sterilized tweezers, and each piece was placed in the bead-beating tube (Lysing Matrix E tube; MP Biomedicals). After DNA extraction following the bead-beating method described previously [72], the 16 DNA samples were mixed and then stored at −80 °C until used. Sequence libraries were prepared with Nextera XT DNA Library Preparation kit (Illumina) with a genomic DNA fragment size ranging from 200 to 2000 bp. These libraries sequenced on HiSeq2500 sequencing platform (Illumina) with HiSeq Rapid SBS kit v2 (Illumina), generating paired-end reads up to 250 bp. The generated sequences were trimmed using Trimmomatic v0.33 [73] with a quality cutoff of 30, sliding window of 6 bp, and minimum length cutoff of 78 bp. The trimmed sequences were assembled using SPAdes v3.10.1 [74] with the “-meta” option and k-mer values of 21, 31, 41, 53, 65, and 77. The assembled contigs were binned using MaxBin2.2.1 [75, 76]. The completeness and contamination of each bin were checked using CheckM [77]. These bins were manually curated as described in our metagenomics study [40]. Genes were then annotated using Prokka v1.12 [78] and eggnog-mapper [79]. For interpretation and comparison of microbial metabolism, bin genomes were also constructed from public metagenomic data generated from The Cedars [3] (trimmed with sliding window of 6, quality cutoff of 20, and a minimum length of 68 bp through Trimmomatic v0.33, normalized using BBMap 36.99 (https://jgi.doe.gov/data-and-tools/bbtools/) with target and minimum coverages of 40 and 2, assembled using SPAdes v3.10.1 with the “-meta” option and k-mer values of 21, 33, 45, 55, 67, and binned through MaxBin2.2.1) and were then analyzed collectively.

### Phylogenomic and phylogenetic analysis

For tree construction, sequences were aligned with MAFFT [80] v7.453 (default parameters) and trimmed using trimAl [81] v1.2rev59 (-gt 0.9) or BMGE v1.12 (-m BLOSUM30) [82]. For ribosomal protein trees, a concatenated alignment of universally conserved ribosomal proteins [83] was used. Protein sequences were retrieved by downloading the GTDB [84] database and predicting protein sequences using Prokka [78] 1.14 (-kingdom Bacteria/Archaea -rnammer -addgenes -mincontiglen 200). Maximum likelihood trees were calculated using IQ-TREE v2.1.3 using the LG [85] model and C20 mixture model with 1000 ultrafast bootstrap replicates (-m LG+C20+G+F -B 1000 -tbe). For ribosomal protein tree calculation with IQ-TREE, a universal distribution mixture (UDM) model with 64 components and LCLR transformation constructed based on the HOGENOM and HSSP databases (-m Poisson+UDM0064LCLR) [86], ultrafast bootstrap approximation (-B 1000), and SH-like approximate likelihood ratio test (-alrt 1000) were used instead. The number of components for the mixture model was selected as the maximum number of components that did not result in estimation of mixture weights close to zero, as indicated by IQ-TREE. The UDM model was chosen over the C60 mixture model often used in ribosomal protein tree calculation as it has been shown to have improved model fit and performance [86, 87]. The model that integrates both the HOGENOM and HSSP databases was used. Bootstrap values were recalculated using BOOSTER [88] (-tbe for IQ-TREE).

For glycine reductase phylogeny, sequence clustering was performing through CD-HIT [89] v4.8.1. For glycine reductase GrdB and sarcosine reductase GrdF, conserved motifs were predicted by first identifying fully conserved residues in the sequence cluster including the biochemically characterized *Peptoclostridium acidaminophilum* GrdF ((YxNx(6)GGE x(34,38) CGD x(27,35) GPxF[NF]AGRYG x(150,181) IHGGYDRx(6)[IP]x(4)PxD x(19,20) TTGTGTx(7)F x(12) [HILV])), then identifying fully conserved residues in the phylogenetic clusters that include GrdB from phyla known to perform the Stickland reaction (*Firmicutes*, *Spirochaetes*, and *Synergistetes*) subtracting any sequences clusters that contain the GrdF motif above (YxNx(6)GGE x(34,38) CGD x(27,35) GPxF[NF]AGRYG x(157,178) AHGGxD[QTAP] x(8) RV[IL]PxD x(19,20) TxGNxTxV)). Ancestral sequence reconstruction for GrdBE was performed using IQ-TREE (-asr), an alignment of glycine reductase sequences included in the concatenated tree (including one archaeal sequence that had mismatches and excluding proline reductases) (MAFFT with default parameters), and a tree rooted with the archaeal GrdBE-like sequence and D-proline reductase subunits PrdBA sequences as the outgroups. The pH preference of the reconstructed sequence was estimated using AcalPred [90].

## Supplementary information

Supplementary Information

## Data Availability

The datasets generated during and/or analyzed during the current study are available in the National Center for Biotechnology Information (NCBI) under BioProject PRJNA453100 and BioSamples SAMN08978938-SAMN08978962.

## References

[1] Brazelton WJ, Thornton CN, Hyer A, Twing KI, Longino AA, Lang SQ (2017). Metagenomic identification of active methanogens and methanotrophs in serpentinite springs of the Voltri Massif, Italy. PeerJ..

[2] Brazelton WJ, Nelson B, Schrenk MO (2011). Metagenomic evidence for H_2_ oxidation and H_2_ production by serpentinite-hosted subsurface microbial communities. Front Microbiol.

[3] Suzuki S, Ishii S, Hoshino T, Rietze A, Tenney A, Morrill PL (2017). Unusual metabolic diversity of hyperalkaliphilic microbial communities associated with subterranean serpentinization at The Cedars. ISME J.

[4] Kelley DS, Karson JA, Früh-Green GL, Yoerger DR, Shank TM, Butterfield DA (2005). A serpentinite-hosted ecosystem: the lost city hydrothermal field. Science..

[5] Tiago I, Verissimo A (2013). Microbial and functional diversity of a subterrestrial high pH groundwater associated to serpentinization. Environ Microbiol.

[6] Crespo-Medina M, Twing KI, Sánchez-Murillo R, Brazelton WJ, McCollom TM, Schrenk MO (2017). Methane dynamics in a tropical serpentinizing environment: The Santa Elena Ophiolite, Costa Rica. Front Microbiol.

[7] Twing KI, Brazelton WJ, Kubo MDY, Hyer AJ, Cardace D, Hoehler TM (2017). Serpentinization-influenced groundwater harbors extremely low diversity microbial communities adapted to high pH. Front Microbiol.

[8] Neubeck A, Sun L, Müller B, Ivarsson M, Hosgörmez H, Özcan D (2017). Microbial community structure in a serpentine-hosted abiotic gas seepage at the Chimaera Ophiolite, Turkey. Appl Environ Microbiol.

[9] Fones EM, Colman DR, Kraus EA, Stepanauskas R, Templeton AS, Spear JR (2021). Diversification of methanogens into hyperalkaline serpentinizing environments through adaptations to minimize oxidant limitation. ISME J.

[10] Schrenk MO, Brazelton WJ, Lang SQ (2013). Serpentinization, carbon, and deep life. Rev Miner Geochem.

[11] Komiya T, Maruyama S, Hirata T, Yurimoto H, Nohda S (2004). Geochemistry of the oldest MORB and OIB in the Isua Supracrustal Belt, southern West Greenland: Implications for the composition and temperature of early Archean upper mantle. Isl Arc.

[12] Sleep NH, Bird DK, Pope EC (2011). Serpentinite and the dawn of life. Philos Trans R Soc Lond B Biol Sci.

[13] Martin WF, Sousa FL (2016). Early microbial evolution: the age of anaerobes. Cold Spring Harb Perspect Biol.

[14] Lane N, Martin, William F (2012). The origin of membrane bioenergetics. Cell.

[15] Battistuzzi FU, Feijao A, Hedges SB (2004). A genomic timescale of prokaryote evolution: insights into the origin of methanogenesis, phototrophy, and the colonization of land. BMC Evol Biol.

[16] Shibuya T, Yoshizaki M, Sato M, Shimizu K, Nakamura K, Omori S (2015). Hydrogen-rich hydrothermal environments in the Hadean ocean inferred from serpentinization of komatiites at 300 °C and 500 bar. Prog Earth Planet Sci.

[17] Camprubi E, Jordan SF, Vasiliadou R, Lane N (2017). Iron catalysis at the origin of life. IUBMB life.

[18] Decker K, Jungermann K, Thauer RK (1970). Energy production in anaerobic organisms. Angew Chem Int Ed Engl.

[19] Martin WF, Weiss MC, Neukirchen S, Nelson-Sathi S, Sousa FL (2016). Physiology, phylogeny, and LUCA. MicrobialCell.

[20] Weiss MC, Sousa FL, Mrnjavac N, Neukirchen S, Roettger M, Nelson-Sathi S (2016). The physiology and habitat of the last universal common ancestor. Nat Microbiol.

[21] Adam PS, Borrel G, Gribaldo S (2018). Evolutionary history of carbon monoxide dehydrogenase/acetyl-CoA synthase, one of the oldest enzymatic complexes. Proc Natl Acad Sci.

[22] Lane N (2017). Proton gradients at the origin of life. Bioessays..

[23] Shibuya T, Russell MJ, Takai K (2016). Free energy distribution and hydrothermal mineral precipitation in Hadean submarine alkaline vent systems: Importance of iron redox reactions under anoxic conditions. Geochim Cosmochim Acta.

[24] Sojo V, Herschy B, Whicher A, Camprubí E, Lane N (2016). The origin of life in alkaline hydrothermal vents. Astrobiology..

[25] Morrill PL, Kuenen JG, Johnson OJ, Suzuki S, Rietze A, Sessions AL (2013). Geochemistry and geobiology of a present-day serpentinization site in California: The Cedars. Geochim Cosmochim Acta.

[26] Suda K, Gilbert A, Yamada K, Yoshida N, Ueno Y (2017). Compound– and position-specific carbon isotopic signatures of abiogenic hydrocarbons from on-land serpentinite-hosted Hakuba Happo hot spring in Japan. Geochim Cosmochim Acta.

[27] Suda K, Ueno Y, Yoshizaki M, Nakamura H, Kurokawa K, Nishiyama E (2014). Origin of methane in serpentinite-hosted hydrothermal systems: the CH_4_–H_2_–H_2_O hydrogen isotope systematics of the Hakuba Happo hot spring. Earth Planet Sci Lett.

[28] Suda K. Origins of hydrocarbons in on-land serpentinization fields and insights into hadean hydrothermal systems: systematic study using stable isotopes. Doctor thesis, Tokyo Institute of Technology. 2016.

[29] Schink B (1997). Energetics of syntrophic cooperation in methanogenic degradation. Microbiol Mol Biol Rev.

[30] Preiner M, Igarashi K, Muchowska KB, Yu M, Varma SJ, Kleinermanns K (2020). A hydrogen-dependent geochemical analogue of primordial carbon and energy metabolism. Nat Ecol Evol.

[31] Suzuki S, Nealson KH, Ishii S (2018). Genomic and in-situ transcriptomic characterization of the candidate phylum NPL-UPL2 from highly alkaline highly reducing serpentinized groundwater. Front Microbiol..

[32] Morandi P, Valzasina B, Colombo C, Curti B, Vanoni MA (2000). Glutamate synthase:  identification of the NADPH-binding site by site-directed mutagenesis. Biochemistry..

[33] Schneider K, Schlegel HG (1976). Purification and properties of soluble hydrogenase from Alcaligenes eutrophus H 16. Biochim Biophys Acta.

[34] Burgdorf T, van der Linden E, Bernhard M, Yuan Yin Q, Back JW, Hartog AF (2005). The soluble NAD^+^-reducing [NiFe]-hydrogenase from *Ralstonia eutropha* H16 consists of six subunits and can be specifically activated by NADPH. J Bacteriol.

[35] de Luca G, de Philip P, Rousset M, Belaich JP, Dermoun Z (1998). The NADP-reducing hydrogenase of *Desulfovibrio fructosovorans*: evidence for a native complex with hydrogen-dependent methyl-viologen-reducing activity. Biochem Biophys Res Commun.

[36] Schut GJ, Adams MW (2009). The iron-hydrogenase of *Thermotoga maritima* utilizes ferredoxin and NADH synergistically: a new perspective on anaerobic hydrogen production. J Bacteriol.

[37] de Bok FA, Hagedoorn PL, Silva PJ, Hagen WR, Schiltz E, Fritsche K (2003). Two W-containing formate dehydrogenases (CO_2_-reductases) involved in syntrophic propionate oxidation by *Syntrophobacter fumaroxidans*. Eur J Biochem.

[38] Yamamoto I, Saiki T, Liu SM, Ljungdahl LG (1983). Purification and properties of NADP-dependent formate dehydrogenase from *Clostridium thermoaceticum*, a tungsten-selenium-iron protein. J Biol Chem.

[39] Hidalgo-Ahumada CAP, Nobu MK, Narihiro T, Tamaki H, Liu WT, Kamagata Y (2018). Novel energy conservation strategies and behaviour of *Pelotomaculum schinkii* driving syntrophic propionate catabolism. Environ Microbiol.

[40] Nobu MK, Narihiro T, Rinke C, Kamagata Y, Tringe SG, Woyke T (2015). Microbial dark matter ecogenomics reveals complex synergistic networks in a methanogenic bioreactor. ISME J.

[41] Poehlein A, Schmidt S, Kaster AK, Goenrich M, Vollmers J, Thurmer A (2012). An ancient pathway combining carbon dioxide fixation with the generation and utilization of a sodium ion gradient for ATP synthesis. PLoS One.

[42] Fuchida S, Mizuno Y, Masuda H, Toki T, Makita H (2014). Concentrations and distributions of amino acids in black and white smoker fluids at temperatures over 200 °C. Org Geochem.

[43] Haberstroh PR, Karl DM (1989). Dissolved free amino acids in hydrothermal vent habitats of the Guaymas Basin. Geochim Cosmochim Acta.

[44] Svensson E, Skoog A, Amend JP (2004). Concentration and distribution of dissolved amino acids in a shallow hydrothermal system, Vulcano Island (Italy). Org Geochem.

[45] Fox SW, Windsor CR (1970). Synthesis of amino acids by the heating of formaldehyde and ammonia. Science..

[46] Islam MN, Kaneko T, Kobayashi K (2001). Determination of amino acids formed in a supercritical water flow reactor simulating submarine hydrothermal systems. Anal Sci.

[47] Inaba S (2018). Primary formation path of formaldehyde in hydrothermal vents. Orig Life Evol Biosph.

[48] Andreesen JR (2004). Glycine reductase mechanism. Curr Opin Chem Biol.

[49] Andreesen JR. Acetate via glycine: a different form of acetogenesis. In: Drake HL, (ed). Acetogenesis. Springer US, Boston, MA. 1994; pp.568–629.

[50] Nisman B (1954). The Stickland reaction. Bacteriol Rev.

[51] Hormann K, Andreesen JR (1989). Reductive cleavage of sarcosine and betaine by *Eubacterium acidaminophilum* via enzyme systems different from glycine reductase. Arch Microbiol.

[52] Moune S, Manac’h N, Hirschler A, Caumette P, Willison JC, Matheron R (1999). *Haloanaerobacter salinarius* sp. nov., a novel halophilic fermentative bacterium that reduces glycine-betaine to trimethylamine with hydrogen or serine as electron donors; emendation of the genus *Haloanaerobacter*. Int J Syst Bacteriol.

[53] Hamdi O, Ben Hania W, Postec A, Bouallagui H, Hamdi M, Bonin P (2015). *Aminobacterium thunnarium* sp. nov., a mesophilic, amino acid-degrading bacterium isolated from an anaerobic sludge digester, pertaining to the phylum Synergistetes. Int J Syst Evol Microbiol.

[54] The UniProt C. (2021). UniProt: the universal protein knowledgebase in 2021. Nucleic Acids Res.

[55] Reimer LC, Vetcininova A, Carbasse JS, Söhngen C, Gleim D, Ebeling C (2019). BacDive in 2019: bacterial phenotypic data for High-throughput biodiversity analysis. Nucleic Acids Res.

[56] Nakai R, Baba T, Niki H, Nishijima M, Naganuma T (2015). *Aurantimicrobium minutum* gen. nov., sp. nov., a novel ultramicrobacterium of the family *Microbacteriaceae*, isolated from river water. Int J Syst Evol Microbiol.

[57] Nakai R, Fujisawa T, Nakamura Y, Nishide H, Uchiyama I, Baba T (2016). Complete genome sequence of *Aurantimicrobium minutum* type strain KNC^T^, a planktonic ultramicrobacterium isolated from river water. Genome Announc.

[58] Giovannoni SJ, Tripp HJ, Givan S, Podar M, Vergin KL, Baptista D (2005). Genome streamlining in a cosmopolitan oceanic bacterium. Science..

[59] Hahn MW, Schmidt J, Taipale SJ, Doolittle WF, Koll U (2014). *Rhodoluna lacicola* gen. nov., sp. nov., a planktonic freshwater bacterium with stream-lined genome. Int J Syst Evol Microbiol.

[60] Suzuki S, Kuenen JG, Schipper K, van der Velde S, Ishii SI, Wu A (2014). Physiological and genomic features of highly alkaliphilic hydrogen-utilizing *Betaproteobacteria* from a continental serpentinizing site. Nat Commun.

[61] Fones EM, Colman DR, Kraus EA, Nothaft DB, Poudel S, Rempfert KR (2019). Physiological adaptations to serpentinization in the Samail Ophiolite, Oman. ISME J.

[62] Mendler K, Chen H, Parks DH, Lobb B, Hug LA, Doxey AC (2019). AnnoTree: visualization and exploration of a functionally annotated microbial tree of life. Nucleic Acids Res.

[63] Kato S, Yumoto I, Kamagata Y (2015). Isolation of acetogenic bacteria that induce biocorrosion by utilizing metallic iron as the sole electron donor. Appl Environ Microbiol.

[64] Morrill PL, Brazelton WJ, Kohl L, Rietze A, Miles SM, Kavanagh H (2014). Investigations of potential microbial methanogenic and carbon monoxide utilization pathways in ultra-basic reducing springs associated with present-day continental serpentinization: the Tablelands, NL, CAN. Front Microbiol.

[65] Chivian D, Brodie EL, Alm EJ, Culley DE, Dehal PS, DeSantis TZ (2008). Environmental genomics reveals a single-species ecosystem deep within Earth. Science.

[66] Lin L-H, Wang P-L, Rumble D, Lippmann-Pipke J, Boice E, Pratt LM (2006). Long-term sustainability of a high-energy, low-diversity crustal biome. Science.

[67] Karnachuk OV, Frank YA, Lukina AP, Kadnikov VV, Beletsky AV, Mardanov AV (2019). Domestication of previously uncultivated *Candidatus* Desulforudis audaxviator from a deep aquifer in Siberia sheds light on its physiology and evolution. ISME J..

[68] Heuer V, Elvert M, Tille S, Krummen M, Mollar XP, Hmelo LR (2006). Online *δ*^13^C analysis of volatile fatty acids in sediment/porewater systems by liquid chromatography‐isotope ratio mass spectrometry. Limnol Oceanogr Methods.

[69] Ijiri A, Harada N, Hirota A, Tsunogai U, Ogawa NO, Itaki T (2012). Biogeochemical processes involving acetate in sub-seafloor sediments from the Bering Sea shelf break. Org Geochem.

[70] Hanselmann KW (1991). Microbial energetics applied to waste repositories. Experientia..

[71] Wang G, Spivack AJ, D’Hondt S (2010). Gibbs energies of reaction and microbial mutualism in anaerobic deep subseafloor sediments of ODP Site 1226. Geochim Cosmochim Acta.

[72] Mason OU, Hazen TC, Borglin S, Chain PS, Dubinsky EA, Fortney JL (2012). Metagenome, metatranscriptome and single-cell sequencing reveal microbial response to Deepwater Horizon oil spill. ISME J.

[73] Bolger AM, Lohse M, Usadel B (2014). Trimmomatic: a flexible trimmer for Illumina sequence data. Bioinformatics..

[74] Bankevich A, Nurk S, Antipov D, Gurevich AA, Dvorkin M, Kulikov AS (2012). SPAdes: a new genome assembly algorithm and its applications to single-cell sequencing. J Comput Biol.

[75] Wu Y-W, Simmons BA, Singer SW (2016). MaxBin 2.0: an automated binning algorithm to recover genomes from multiple metagenomic datasets. Bioinformatics..

[76] Wu YW, Tang YH, Tringe SG, Simmons BA, Singer SW (2014). MaxBin: an automated binning method to recover individual genomes from metagenomes using an expectation-maximization algorithm. Microbiome..

[77] Parks DH, Imelfort M, Skennerton CT, Hugenholtz P, Tyson GW (2015). CheckM: assessing the quality of microbial genomes recovered from isolates, single cells, and metagenomes. Genome Res.

[78] Seemann T (2014). Prokka: rapid prokaryotic genome annotation. Bioinformatics..

[79] Huerta-Cepas J, Szklarczyk D, Forslund K, Cook H, Heller D, Walter MC (2016). eggNOG 4.5: a hierarchical orthology framework with improved functional annotations for eukaryotic, prokaryotic and viral sequences. Nucleic Acids Res.

[80] Katoh K, Kuma K-I, Toh H, Miyata T (2005). MAFFT version 5: improvement in accuracy of multiple sequence alignment. Nucleic Acids Res.

[81] Capella-Gutiérrez S, Silla-Martínez JM, Gabaldón T (2009). trimAl: a tool for automated alignment trimming in large-scale phylogenetic analyses. Bioinformatics..

[82] Criscuolo A, Gribaldo S (2010). BMGE (Block Mapping and Gathering with Entropy): a new software for selection of phylogenetic informative regions from multiple sequence alignments. BMC Evol Biol.

[83] Imachi H, Nobu MK, Nakahara N, Morono Y, Ogawara M, Takaki Y (2020). Isolation of an archaeon at the prokaryote-eukaryote interface. Nature..

[84] Parks DH, Chuvochina M, Chaumeil P-A, Rinke C, Mussig AJ, Hugenholtz P (2020). A complete domain-to-species taxonomy for bacteria and archaea. Nat Biotechnol.

[85] Le SQ, Gascuel O (2008). An improved general amino acid replacement matrix. Mol Biol Evol.

[86] Schrempf D, Lartillot N, Szöllősi G (2020). Scalable empirical mixture models that account for across-site compositional heterogeneity. Mol Biol Evol.

[87] Williams TA, Schrempf D, Szöllősi GJ, Cox CJ, Foster PG, Embley TM (2021). Inferring the deep past from molecular data. Genome Biol Evol.

[88] Lemoine F, Domelevo Entfellner JB, Wilkinson E, Correia D, Dávila Felipe M, De Oliveira T (2018). Renewing Felsenstein’s phylogenetic bootstrap in the era of big data. Nature..

[89] Fu L, Niu B, Zhu Z, Wu S, Li W (2012). CD-HIT: accelerated for clustering the next-generation sequencing data. Bioinformatics.

[90] Lin H, Chen W, Ding H (2013). AcalPred: a sequence-based tool for discriminating between acidic and alkaline enzymes. PLoS One.

