# Supplementary material for: Unique H2-utilizing lithotrophy in serpentinite-hosted systems
Source: ISME J. 2022 Oct 7;17(1):95–104. doi: 10.1038/s41396-022-01197-9 (PMC9751293; doi:10.1038/s41396-022-01197-9)
Supplement: Supplementary file 1 — Supplementary Information [file 41396_2022_1197_MOESM1_ESM.pdf]

Unique H_2-utilizing lithotrophy in serpentinite-hosted systems

- Supplementary Information -

Masaru Konishi Nobu[1†*], Ryosuke Nakai[1,2†], Satoshi Tamazawa[1,3],
Hiroshi Mori[4], Atsushi Toyoda[4], Akira Ijiri[5], Shino Suzuki[6,7],
Ken Kurokawa[4], Yoichi Kamagata[1], and Hideyuki Tamaki[1*]

Affiliation:
[1] Bioproduction Research Institute, National Institute of Advanced Industrial Science and Technology (AIST), 1-1-1 Higashi, Tsukuba, Ibaraki 305-8566, Japan
[2] Bioproduction Research Institute, National Institute of Advanced Industrial Science and Technology (AIST), 2-17-2-1, Tsukisamu-Higashi, Sapporo, 062-8517, Japan
[3] Horonobe Research Institute for the Subsurface Environment (H-RISE), Northern Advancement Center for Science & Technology, 5-3 Sakaemachi, Horonobe, Teshio, Hokkaido, 098-3221, Japan
[4] National Institute of Genetics, 1111 Yata, Mishima, Shizuoka 411-8540, Japan
[5] Kochi Institute for Core Sample Research, Japan Agency for Marine-Earth Science and Technology (JAMSTEC), 200 Monobe Otsu, Nankoku, Kochi, Japan
[6] Institute for Extra-cutting-edge Science and Technology Avant-garde Research (X-star), JAMSTEC, Natsushima 2-15, Yokosuka, Kanagawa 237-0061, Japan
[7] Institute of Space and Astronautical Science (ISAS), Japan Aerospace Exploration Agency (JAXA), 3-1-1 Yoshinodai, Chuo-ku, Sagamihara, Kanagawa 252-5210, Japan

[†] These authors contributed equally.
* Corresponding author: m.nobu@aist.go.jp and tamaki-hideyuki@aist.go.jp

Table of Contents

Supplementary Results 2

Supplementary Figures 4
  Figure S1 4
  Figure S2 5
  Figure S3 6
  Figure S4 7
  Figure S5 8
  Figure S6 9
  Figure S7 10
  Figure S8 11
  References 12

Supplementary Equations.................. 13

Supplementary Tables 14
  Table S1 14
  Table S2 15
  Table S3 16
  Table S4 17
  Table S5 18
  Table S6 19

H_2 and formate metabolism

Assuming that the hydrogenases and formate dehydrogenases *in situ* use NADP(H) or NAD(H)+ferredoxin (*i.e.*, electron-bifurcating) (an assumption confirmed based on analysis of the metagenome-assembled genomes we recover; see below), H_2 and formate are likely reductants. In Hakuba, we estimate ΔG of +8.64 and +4.78 kJ per mol H_2 for H_2 generation through the respective pathways, assuming (i) literature cytosolic electron carrier redox potentials (-370 mV for NADPH, -320 mV NADH, and -450 mV Fd), (ii) cytosolic pH of 8.8 (two units lower than extracellular milieu (Krulwich et al 2011)), and (iii) intracellular H_2 concentrations similar to surrounding environment. As for formate metabolism, formate dehydrogenases are predicted to run in the oxidative direction because CO_2 reduction to formate is also endergonic in situ (ΔG of +30.28 and +24.92 kJ per mol formate depending on the electron carrier, with identical assumptions). H_2 and formate generation can only become exergonic ($\Delta G > 0$) if cytosolic H_2 and formate reach below 266 nM and 0.115 nM respectively. Similarly, in The Cedars (estimated cytosolic pH of 9.9), H_2 and formate must be less than 20.6 nM and 5.18 nM respectively.

For H_2 metabolism, we identify putative NADP-reducing hydrogenases (HoxEFUHY in *Actinobacteria* and "*Ca*. Lithacetigenota" and HndABCD in Firmicutes) and NAD/Fd-dependent electron-confurcating hydrogenases (HydABC in Firmicutes) (Burgdorf et al 2005, de Bok et al 2003, de Luca et al 1998, Hidalgo-Ahumada et al 2018, Morandi et al 2000, Schneider and Schlegel 1976, Schut and Adams 2009, Yamamoto et al 1983). HoxEFUHY typically uses NAD(H) as an electron carrier, but the Hox-related hydrogenases of HKB210 and BS525 consistently associate with a sixth subunit containing a putative NADPH-binding GltD domain (tentatively HoxP), suggesting that these hydrogenases may employ NADP(H) as an electron carrier rather than NAD(H), a phenomenon that has also been reported for the *Ralstonia eutropha* HoxEFUHYI. For formate metabolism, we predict NADP-dependent formate dehydrogenases in one *Syntrophomonadaceae* population and putative electron-confurcating formate dehydrogenases in *Actinobacteria*, NPL-UPA2, and a *Syntrophomonadaceae* population.

As many putative H_2-utilizers *in situ* depend on the NADP-reducing hydrogenase above, a redox protein for transferring electrons from NADPH to NAD^+ and ferredoxin is necessary for driving the Wood-Ljungadhl pathway (catabolism), Rnf (energy via vectorial cation extrusion), and/or biosynthesis. Several genomes encode the NADH-dependent ferredoxin:$NADP^+$ reductase (NfnAB) for interconversion of these electron carriers, which allows for energy recovery from H_2-/formate-driven homoacetogenesis by *Actinobacteria* and *Syntrophomonadaceae* members (Fig. S5). *Ca.* Lithacetigenota and NPL-UPA2 encode homoacetogenesis from H_2 and/or formate but lack NfnAB. Analysis of potential redox complexes encoded in these genomes revealed a gene cassette of a HydB homolog, a HydC homolog, and a putative NADPH-binding redox protein with a GltD domain highly similar to HoxP (>45% amino acid sequence identity) (Table S5). Given that the function of HydBC is to hand off electrons from an electron-donating protein (typically FeFe hydrogenase HydA) to NAD^+ and ferredoxin, we speculate that the putative NADPH-binding redox protein likely oxidizes NADPH and transfers electrons to NAD^+ and ferredoxin, much like NfnAB. Identification of a flavin-binding site that would support the possibility of flavin-mediated electron bifurcation would be valuable, but, no such flavin-binding site could be identified at this point (as has also been reported for the well-known electron-bifurcating hydrogenase HydABC).

Etymology of candidate taxa

Description of *Candidatus* Lithacetigenota phy. nov.

Lithacetigenota (Lith.a.ce.ti.ge.no'ta. N.L. fem. n. *Lithacetigena*, a candidatus genus name; *-ota*, ending to denote a phylum; N.L. neut. pl. n. *Lithacetigenota*, the *Candiatus* Lithacetigena phylum).

The *Candidatus* Lithacetigenota is defined by five metagenome bins (HKB111, HKB210, BS525, BS5B28, and GPS1B18) recovered using culture-independent metagenomics from two serpentinite-hosted systems (Hakuba Happo hot springs in Hakuba, Japan, and The Cedars springs in California, USA). The metagenome bins are deposited in the National Center for Biotechnology Information (NCBI) under BioProject number PRJNA453100 and WGS numbers QLUP00000000, QLUQ00000000, QLTW00000000, QLTX00000000, and QLTY00000000. See also Table S3 for the quality and completeness of each bin-genome. Genomic analysis predicts that *Ca.* Lithacetigenota bacteria have the capacity to produce acetate from inorganic substrates.

Description of *Candidatus* Lithacetigena gen. nov.

Lithacetigena (Lith.a.ce.ti'ge.na. Gr. masc. n. *lithos*, stone; N.L. neut. n. *acidum aceticum*, acetic acid; L. suff. *-genus -a -um* (from L. v. *gigno*) producing; N.L. fem. n. *Lithacetigena*, producing acetate from inorganic substrate).

The type species is *Candidatus* Lithacetigena glycinireducens with two metagenome bins (HKB111 and HKB210) recovered using metagenomics from Hakuba Happo hot springs in Hakuba, Japan.

Description of *Candidatus* Lithacetigena glycinireducens sp. nov.

Lithacetigena glycinireducens (gly.ci.ni.re.du'cens. N.L. neut. n. *glycinum*, glycine; L. pres. part. *reducens*, bringing back, leading back; N.L. part. adj. *glycinireducens*, glycine-reducing).

The genome of this candidatus species was discovered in Hakuba Happo hot springs in Hakuba, Japan.

Description of *Candidatus* Psychracetigena formicireducens gen. nov.

Psychracetigena (Psychr.a.ce.ti'ge.na. Gr. masc. adj. *psychros*, cold; N.L. neut. n. *acidum aceticum*, acetic acid; L. suff. *-genus -a -um* (from L. v. gigno) producing; N.L. fem. n. *Psychracetigena* producing acetate under psychrophilic conditions).

The type species is *Candidatus* Psychracetigena formicireducens with three metagenome bins (BS525, BS5B28, and GPS1B18) recovered using metagenomics from The Cedars springs in California, USA.

Description of *Candidatus* Psychracetigena formicireducens sp. nov.

Psychracetigena formicireducens (for.mi.ci.re.du'cens. N.L. neut. n. *acidum formicum*, formic acid; L. pres. part. *reducens*, bringing back, leading back; N.L. part. adj. *formicireducens*, formate-reducing).

The genome of this candidatus species was discovered in The Cedars springs in California, USA.

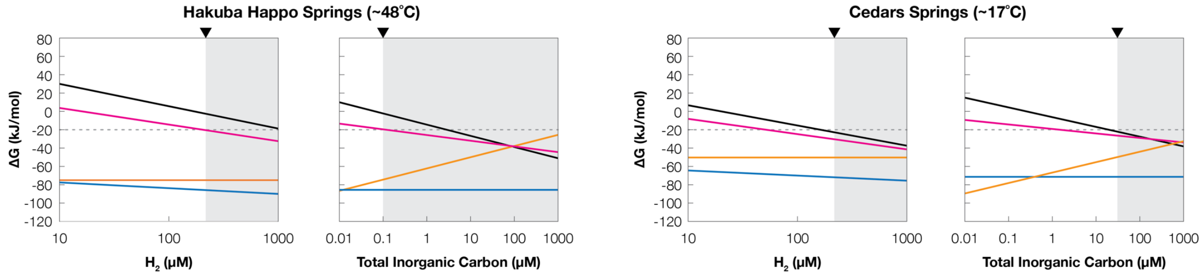

Figure S1. Thermodynamics of H_2-oxidizing reduction of CO_2 (black), formate (pink), and glycine (blue), and formate disproportionation (orange) with changing H_2 and total inorganic carbon (TIC) concentrations. The maximum observed H_2 concentration in Hakuba Happo hot springs (664 μM) is marked (triangles) in the H_2-based plots and the TIC concentrations observed in Hakuba (<0.1 μM) and The Cedars GPS1 (35 μM) are indicated correspondingly. See also Tables S1 and S2 and Supplementary Equations.

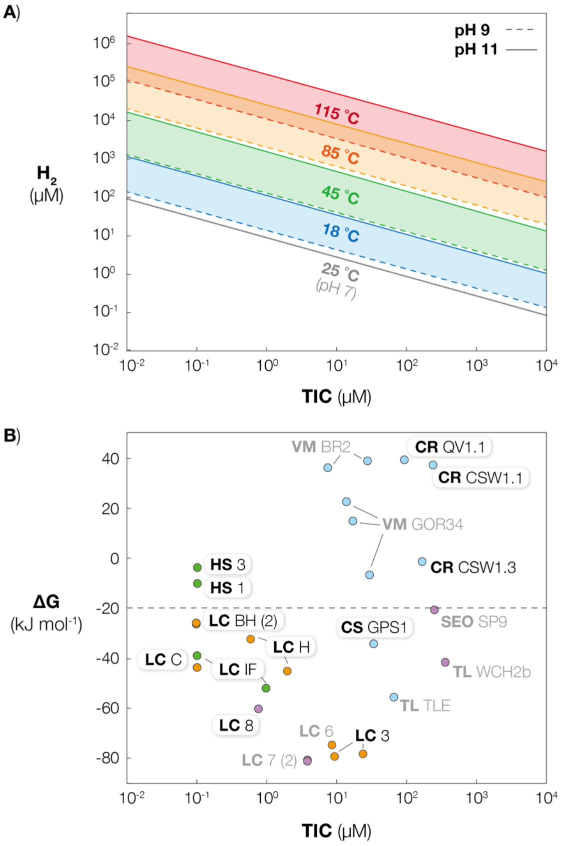

Figure S2. Thermodynamics of H_2-oxidizing CO_2-reducing homoacetogenesis under differing pH, temperature, H_2, and total inorganic carbon (TIC) concentrations. (A) For temperatures 18 (blue), 45 (green), 85 (orange), and 115 (red) °C, the H_2 and TIC concentrations at which H_2/CO_2 homoacetogenesis has a Gibbs free energy yield (ΔG) of -10 kJ mol^{-1} is shown for pH of 9 (dotted line) and 11 (solid line) (atmospheric pressure of 1 atm). For reference, the same is shown for 25 °C at pH 7 (gray solid line). (B) The ΔG of H_2/CO_2 homoacetogenesis in various serpentinite-hosted systems (Hakuba Happo hot springs - HS; The Cedars springs - CS; Lost City - LC; Voltri Massif - VM; Coast Range Ophiolite Microbiological Observatory - CR; Santa Elena ophiolite - SEO; Table Lands - TLE) are shown based on reported environmental conditions for individual sampling locations. Each data point is colored based on temperature: psychrophilic (blue), mesophilic (purple), thermophilic (green), and hyperthermophilic (orange). Samples with associated acetate measurements are labelled black, and those that have >2 µM acetate are circled. For samples with no reported acetate concentrations (gray), the average of reported concentrations was used (8.57 µM Acetate). For The Cedars spring sample, no H_2 concentration has been reported, so the highest on-land serpentinite-hosted system H_2 concentration was used (664 µM H_2 from Hakuba Happo #1). Thermodynamic calculations were performed using $\Delta G°_f$ and $\Delta H°_f$ values at 298 K values and temperature adjustment through the Gibbs-Helmholtz equation. The effect of pressure was approximated as described by Wang *et al*. (Wang et al 2010). See also Tables S1 and S2 and Supplementary Equations.

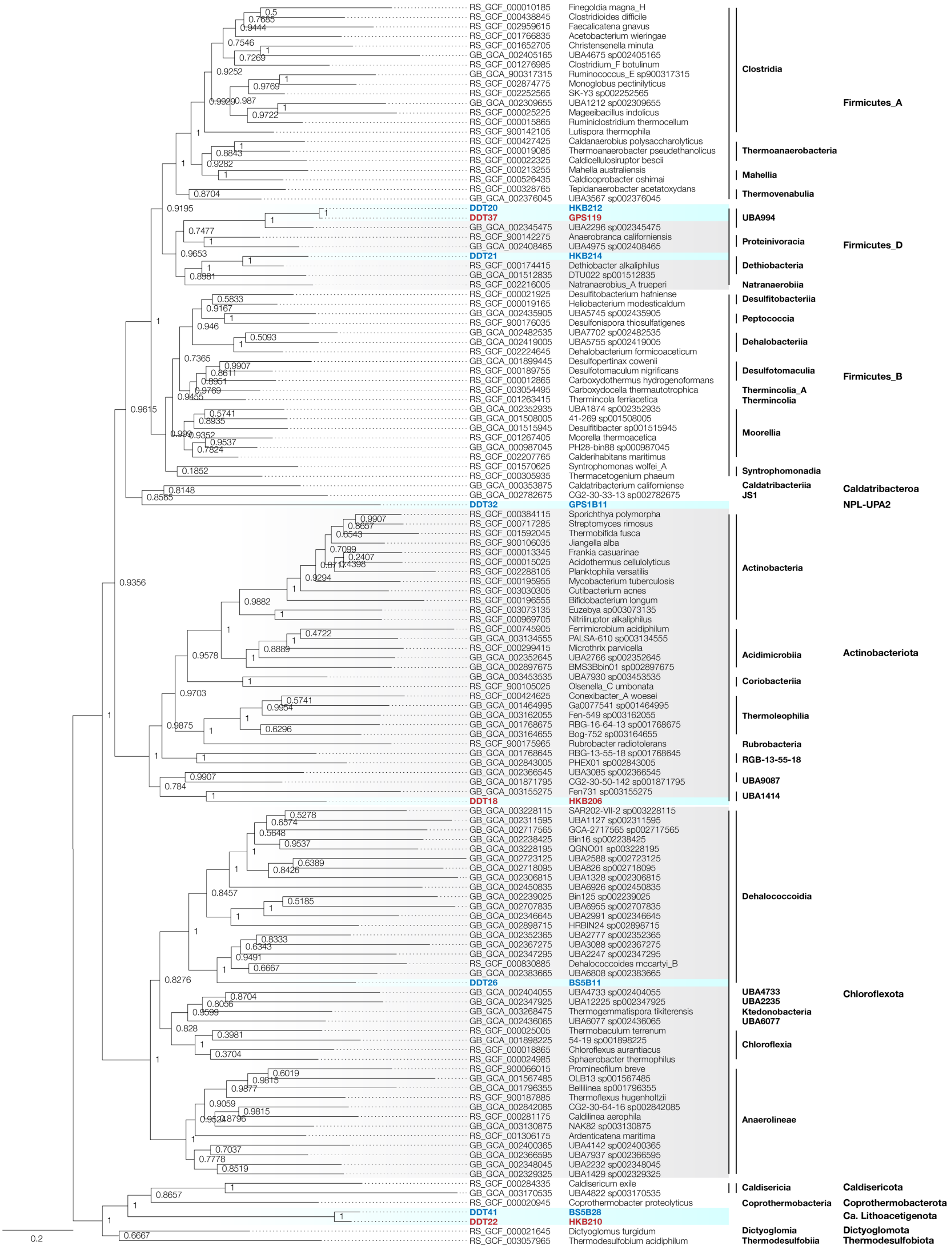

Figure S3. Ribosomal protein tree for high-quality MAGs. Universally conserved ribosomal proteins were collected from each genome, aligned with MAFFT v7.394 (Katoh et al 2005), trimmed with trimAl 1.2rev59 (-gt 0.70) (Capella-Gutiérrez et al 2009), and concatenated. A maximum likelihood tree was calculated using phyML 3.3.20190321 with the LG model and 100 bootstrap iterations (Guindon and Gascuel 2003). GTDBtk-based phylogeny is shown.

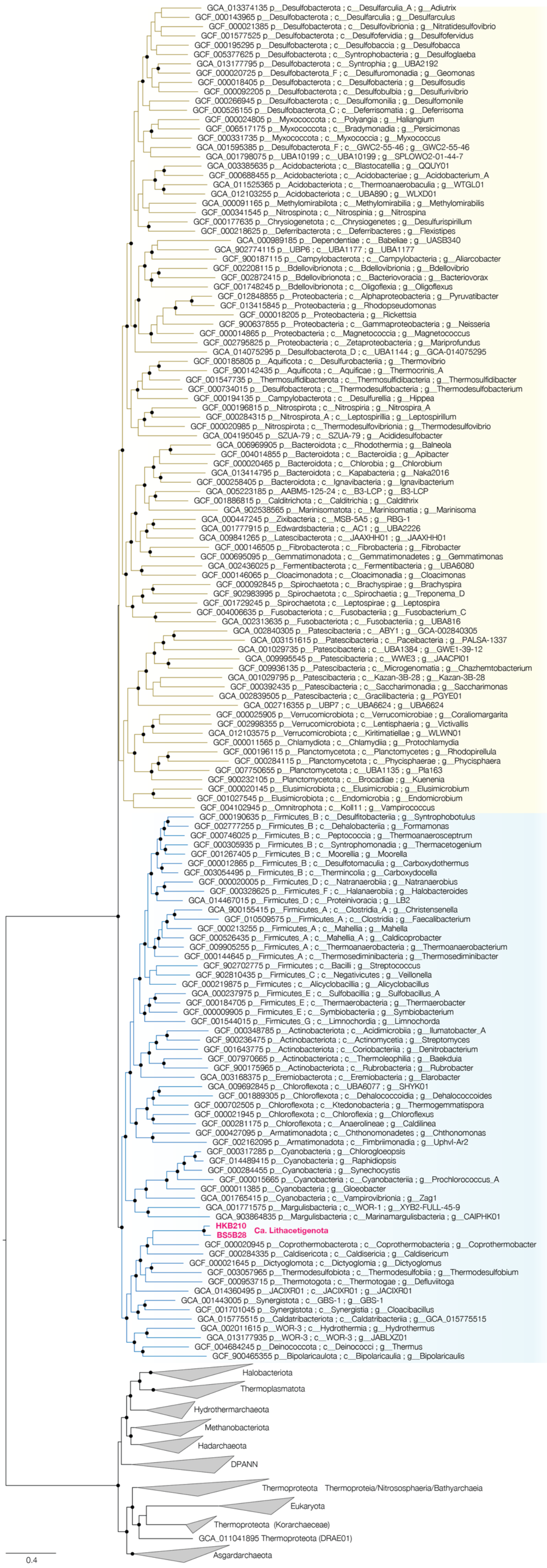

Figure S4. Ribosomal protein tree including high-quality MAGs from 74 GTDB-defined phylum-level lineages. See Fig. 1 for details. GTDBtk-based phylogenetic nomenclature is shown.

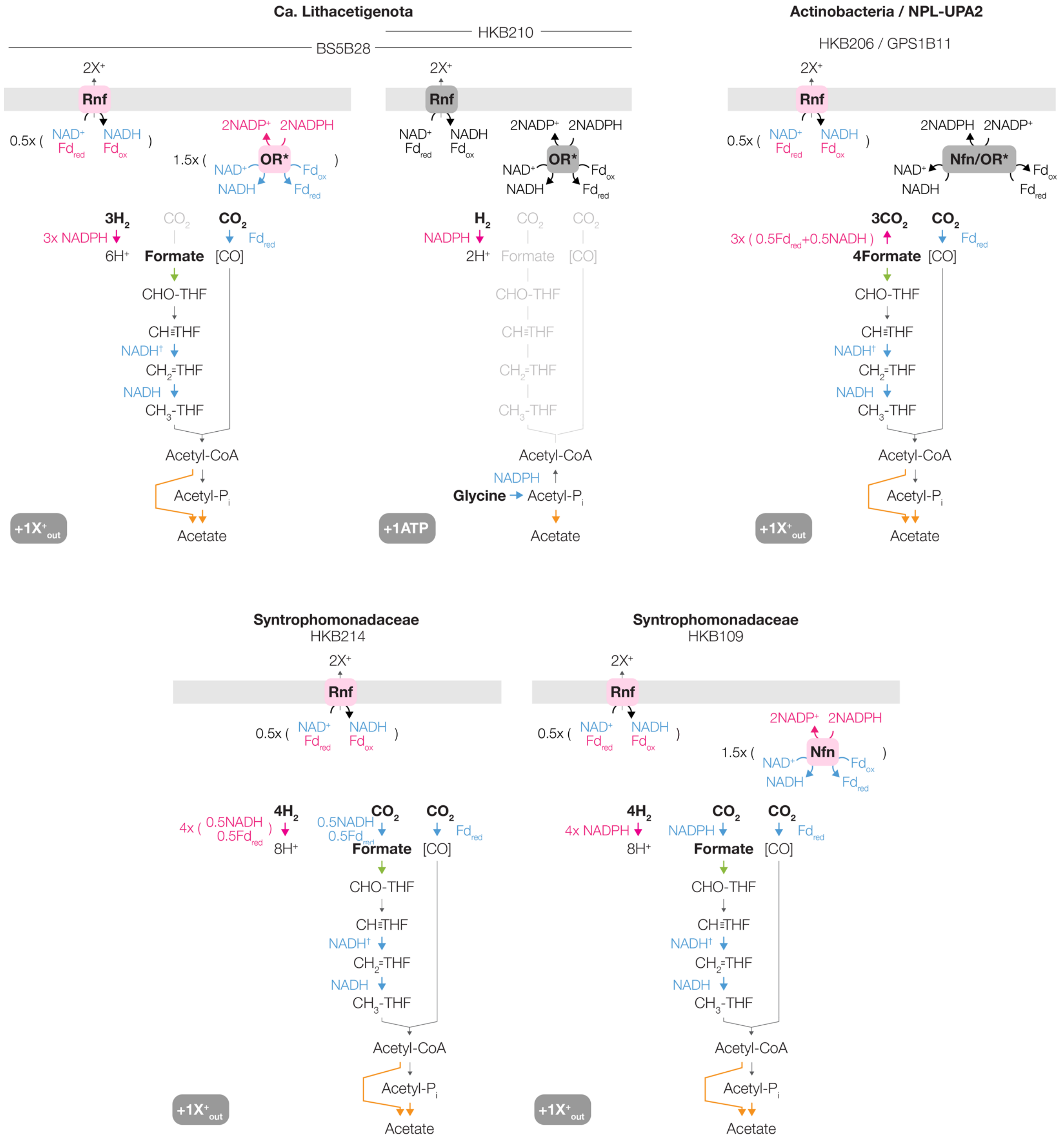

Figure S5. Schemes for energy metabolism. Arrow colors indicate oxidative (pink), reductive (blue), ATP-yielding (orange), and ATP-consuming (green) steps. The corresponding electron carrier (reduced form) is shown for each redox step. Redox reactions shown in gray are necessary for generating reducing equivalents for biosynthesis (e.g., if the putative catabolic pathway does not produce NADPH, an NADPH-generating pathway is shown in gray). Abbreviations: NAD – nicotinamide adenine dinucleotide, NADP – nicotinamide adenine dinucleotide phosphate, Fd – ferredoxin, THF – tetrahydrofolate, CoA – Coenzyme A, Rnf – Ion-translocating NADH:ferredoxin oxidoreductase, Nfn – NADH-dependent ferredoxin:$NADP^+$ oxidoreductase, *OR – putative NADH-dependent NADPH:ferredoxin oxidoreductase (see supplemental results). †For the 5,10-methylene-tetrahydrofolate dehydrogenase reaction, NAD^+/NADH is shown as the electron carrier as has been observed for *Acetobacterium woodii*.

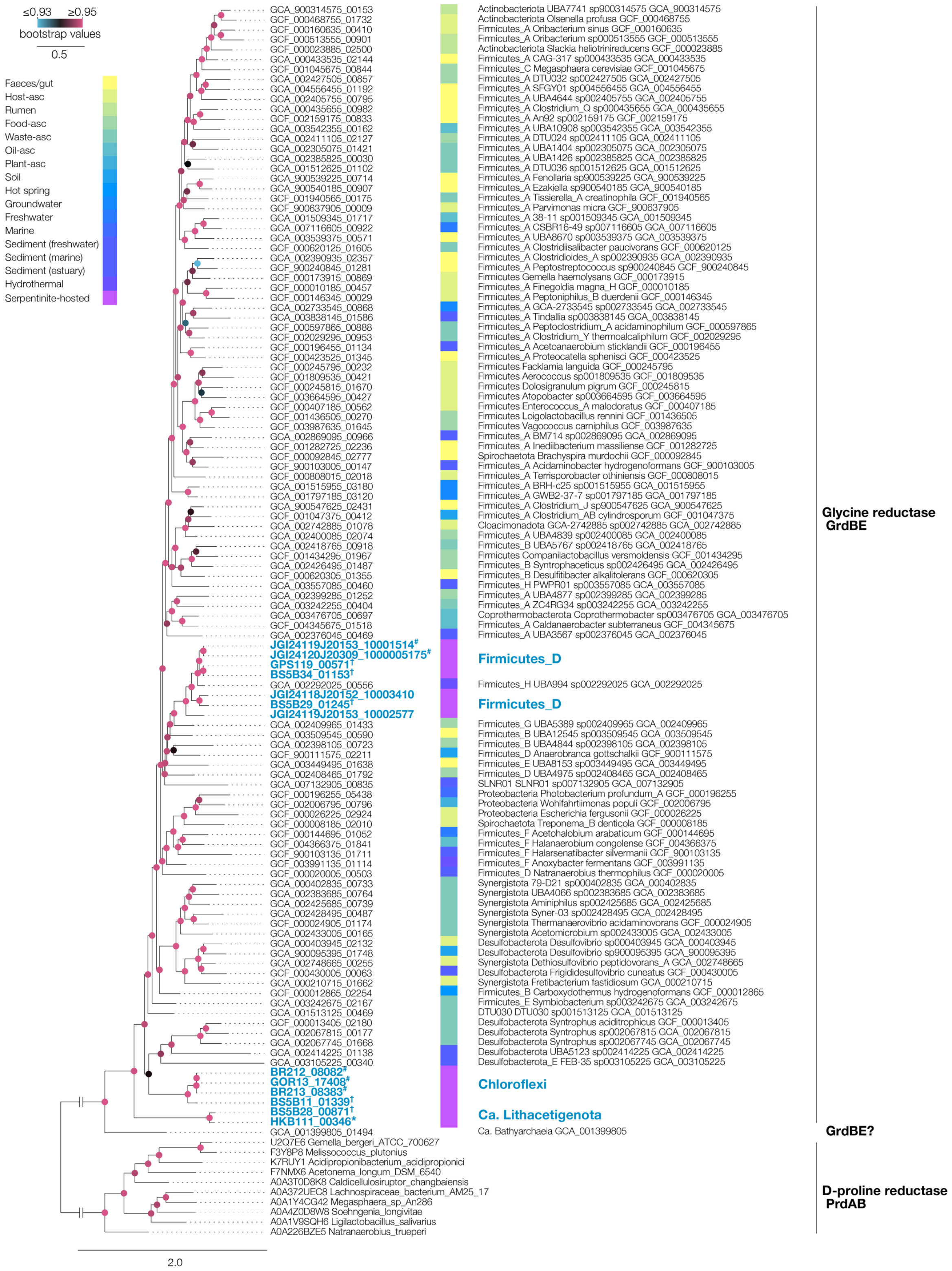

Figure S6. Complete phylogenetic tree of glycine reductase subunits GrdBE and homologs from Hakuba Happo hot spring*, The Cedars springs†, and other serpentinite-hosted system metagenomes#. See Fig. 3 for details. Phylogenetic nomenclature indicated with an asterisk are GTDB-defined phylum-level nomenclature. For those with underscores, the name is abbreviated to the last capital letter (e.g., Firmicutes_A is shown as (A) and Desulfobacterota_E is shown as (E)).

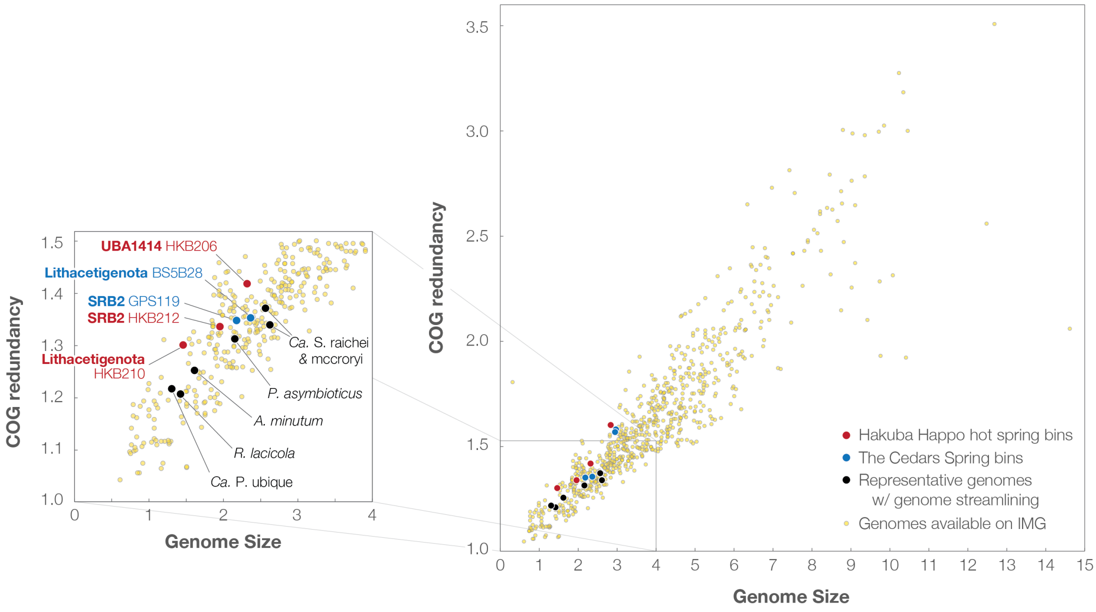

Figure S7. Genome streamlining of publically available genomes (Joint Genome Institute Integrated Microbial Genome) and selected high completeness bins from Hakuba Happo hot springs (red) and The Cedars springs (blue) (inset on left). Genomes with known streamlining are marked (black): *Aurantimicrobium minutum*, *Ca.* Pelagibacter ubique, *Polynucleobacter asymbioticus*, *Rhodoluna lacicola*, *Ca.* Serpentinomonas raichei, and *Ca.* S. mccroryi.

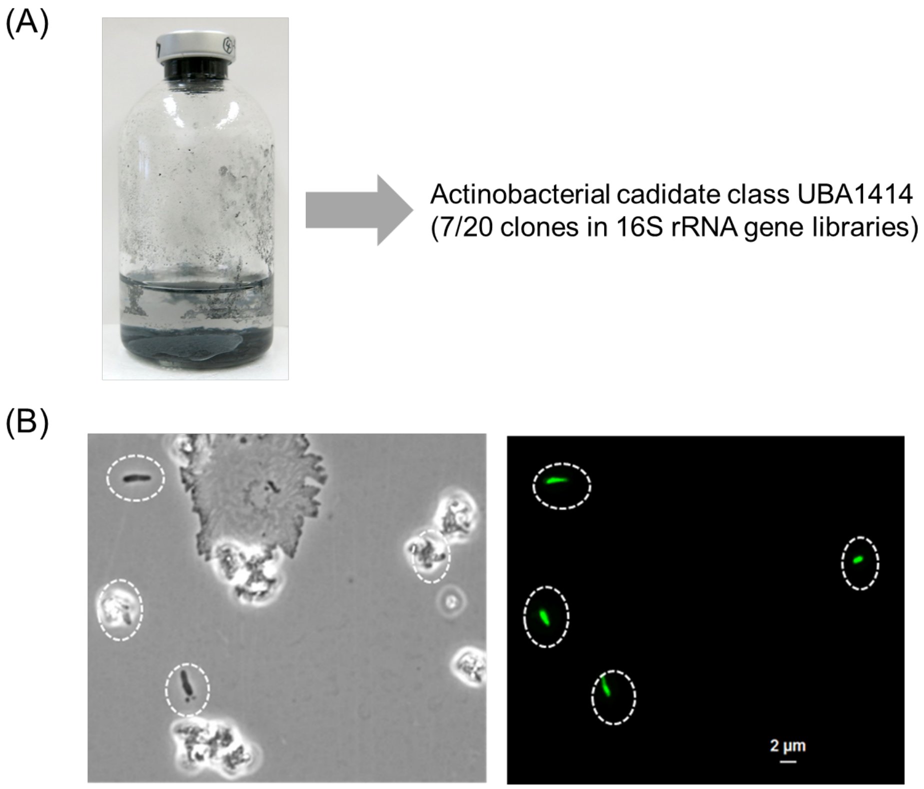

Figure S8. Hakuba-derived enrichment culture of *Actinobacteria* UBA1414. (A) Culture medium based on Widdel medium (pH 10) with an N_2-CO_2 (80:20, vol/vol) headspace was supplemented with 0.01 g l^{-1} yeast extract and 25 g l^{-1} elemental iron granules. Hakuba hot spring water 100 mL was passed though a membrane filter and the filter was submerged in the culture medium. After a 4 month incubation at 40 °C, 1 mL of the culture was used for DNA extraction, PCR amplification, and clone library construction (about 600 bp of 16S rRNA gene). UBA1414-derived 16S rRNA gene fragments, which shared high sequence identity (>99%) to bin HKB206, comprised 7 out of 20 clones. The remaining 13 clones consisted of obligately aerobic *Methylobacterium*- and *Pseudomonas*-related sequences that may be contaminants, but further investigation is required. (B) Micrographs: phase-contrast (left) and SYBR-Green-I-stained microbial cells (green, right); scale bar, 2 µm.

References

Burgdorf T, van der Linden E, Bernhard M, Yuan Yin Q, Back JW, Hartog AF *et al* (2005). The Soluble NAD$^+$-Reducing [NiFe]-Hydrogenase from *Ralstonia eutropha* H16 Consists of Six Subunits and Can Be Specifically Activated by NADPH. *J Bacteriol* **187:** 3122-3132.

Capella-Gutiérrez S, Silla-Martínez JM, Gabaldón T (2009). trimAl: a tool for automated alignment trimming in large-scale phylogenetic analyses. *Bioinformatics* **25:** 1972-1973.

de Bok FA, Hagedoorn PL, Silva PJ, Hagen WR, Schiltz E, Fritsche K *et al* (2003). Two W-containing formate dehydrogenases (CO_2-reductases) involved in syntrophic propionate oxidation by *Syntrophobacter fumaroxidans*. *European journal of biochemistry / FEBS* **270:** 2476-2485.

de Luca G, de Philip P, Rousset M, Belaich JP, Dermoun Z (1998). The NADP-reducing hydrogenase of *Desulfovibrio fructosovorans*: evidence for a native complex with hydrogen-dependent methyl-viologen-reducing activity. *Biochemical and biophysical research communications* **248:** 591-596.

Guindon S, Gascuel O (2003). A simple, fast, and accurate algorithm to estimate large phylogenies by maximum likelihood. *Syst Biol* **52:** 696-704.

Hidalgo-Ahumada CAP, Nobu MK, Narihiro T, Tamaki H, Liu WT, Kamagata Y *et al* (2018). Novel energy conservation strategies and behaviour of *Pelotomaculum schinkii* driving syntrophic propionate catabolism. *Environ Microbiol* **20:** 4503-4511.

Katoh K, Kuma K-i, Toh H, Miyata T (2005). MAFFT version 5: improvement in accuracy of multiple sequence alignment. *Nucleic Acids Res* **33:** 511-518.

Krulwich T, Liu J, Morino M, Fujisawa M, Ito M, B. Hicks D (2011). Adaptive Mechanisms of Extreme Alkaliphiles. In: Horikoshi K (ed). *Extremophiles Handbook*. Springer: Tokyo. pp 119-139.

Morandi P, Valzasina B, Colombo C, Curti B, Vanoni MA (2000). Glutamate synthase: identification of the NADPH-binding site by site-directed mutagenesis. *Biochemistry-Us* **39:** 727-735.

Schneider K, Schlegel HG (1976). Purification and properties of soluble hydrogenase from Alcaligenes eutrophus H 16. *Biochimica et biophysica acta* **452:** 66-80.

Schut GJ, Adams MW (2009). The iron-hydrogenase of *Thermotoga maritima* utilizes ferredoxin and NADH synergistically: a new perspective on anaerobic hydrogen production. *J Bacteriol* **191:** 4451-4457.

Wang G, Spivack AJ, D'Hondt S (2010). Gibbs energies of reaction and microbial mutualism in anaerobic deep subseafloor sediments of ODP Site 1226. *Geochim Cosmochim Ac* **74:** 3938-3947.

Yamamoto I, Saiki T, Liu SM, Ljungdahl LG (1983). Purification and properties of NADP-dependent formate dehydrogenase from Clostridium thermoaceticum, a tungsten-selenium-iron protein. *J Biol Chem* **258:** 1826-1832.

Supplementary Equations

Gibbs-Helmholtz equation:

$$\Delta G_1 = R \times T_1 \times \left(\frac{\Delta G_2}{R \times T_2} - ln\left(\frac{K_{eq1}}{K_{eq2}} \right) \right)$$

van't Hoff equation:

$$ln\left(\frac{K_{eq2}}{K_{eq1}} \right) = \left(\frac{\Delta H}{R} \right) \times \left(\frac{1}{T_1} - \frac{1}{T_2} \right)$$

Pressure adjustment equation (optional):

$$\Delta G_1 = R \times T_1 \times \left(\frac{\Delta G_2 + \Delta V \times (P_1 - P_2)}{R \times T_2} - ln\left(\frac{K_{eq1}}{K_{eq2}} \right) \right)$$

ΔG_1 : Gibbs free energy of the reaction at *in situ* temperature
ΔG_2 : Gibbs free energy of the reaction at the reference temperature
R : Universal gas constant
T_1 : *In situ* temperature (Kelvin)
T_2 : Reference temperature (Kelvin)
ΔH : Molar enthalpy change of the reaction at the reference temperature
K_{eq1} : Equilibrium constant at *in situ* temperature
K_{eq2} : Equilibrium constant at the reference temperature
ΔV : Molar volume change in the reaction at the reference temperature and pressure
P_1 : *In situ* pressure (atm)
P_2 : Reference pressure (atm)

Table S1. Gibbs free energy ($G°_f$) and enthalpy ($H°_f$) of formation of compounds of interest.

Compound name	Formula	State	$G°_{f\,(298)}$ [kJ mol^{-1}]*	$H°_{f\,(298)}$ [kJ mol^{-1}]*	Vm (cm^3 mol^{-1})
Hydrogen	H_2	g	0	0	26.7
Carbon dioxide	CO_2	g	-394.4	-393.5	33.9
Carbon monooxide	CO	g	-137.2	-110.5	37.3
Acetate	CH_3COOH	ionized, aq	-369.4	-486.0	56.3
Formate	HCOOH	ionized, aq	-351.0	-425.6	37.7
Glycine-†	NH_2CH_2COO-	ionized, aq	-325.7	-521.17	64.7
Ammonia	NH_3	aq	-26.6	-80.3	26.56
Water	H_2O	l	-237.2	-285.8	18.03

*Data from Hanselman K.W. Microbial energetics applied to waste repositories. Experientia, 47, 645–687, (1991). https://doi.org/10.1007/BF01958816

†For glycine- only, the $G°_{f\,(298)}$ reported in Amend & Shock (2001) was adjusted using the pKa value (9.6) of the amine group of glycine.

Amend J.P. & Shock E.L. Energetics of overall metabolic reactions of thermophilic and hyperthermophilic Archaea and bacteria. FEMS Microbiol Rev., 25, 175-243, (2001). https://10.1111/j.1574-6976.2001.tb00576.x

Table S2. Environmental parameters and concentration information used for Gibbs free energy yield (ΔG) calculation of H_2/CO_2 homoacetogenesis in various serpentinite-hosted systems (see also Supplementary Equations).

Ecosystm	Location	Sample	Sample code in Figure S2B	*p*H	Temp (°C)	H_2 (μM)[†]	TIC (μM)[‡]	CO_2 (μM)[‡]	Acetate (μM)[§]	ΔG_r[¶]	ΔG_{temp}[¶]	$\Delta G_{temp+prs}$[¶]	Reference
Hakuba Happo	Japan	#1	HS 1	10.8	52	664	0.1	3.38E-07	6	-20.47	2.18	-10.29	1,2,3
Hakuba Happo	Japan	#3	HS 3	10.7	48	201	0.1	5.71E-07	4	-11.39	8.60	-3.71	1,2,3
The Cedars	USA	GPS1	CS GPS1	11.9	17	664	35	2.26E-06	69.3	-27.54	-34.06	-	4, this study for the acetate conc.
Voltri Massif	Italy	GOR34-spring3-2012	VM GOR34	11.8	18.5	26.8	29	2.82E-06	8.57	-1.54	-7.40	-	5,6
Voltri Massif	Italy	BR2-spring-2012	VM BR2	12.1	20.3	0.5	29	6.78E-07	8.57	43.15	38.21	-	5,6
Voltri Massif	Italy	BR2-spring-2013	VM BR2	12.3	20.3	1.8	7.8	7.29E-08	8.57	40.36	35.46	-	5,6
Voltri Massif	Italy	GOR34-spring1-2013	VM GOR34	12.3	18.5	9.2	17.2	1.70E-07	8.57	20.13	13.79	-	5,6
Voltri Massif	Italy	GOR34-spring1-2011	VM GOR34	12.2	18.5	3.9	14	2.19E-07	8.57	27.96	21.45	-	5,6
Table Lands	Canada	TLE	TL TLE	10.6	9.1	180	69.2	1.59E-03	8.57	-44.17	-56.24	-	7
Table Lands	Canada	WHC2b	TL WHC2b	12.3	16.2	520	370.8	3.95E-06	8.57	-35.27	-42.22	-	7
CROMO*	USA	QV1.1	CR QV1.1	11.5	17.9	0.075	96	3.70E-05	10.2	46.19	38.65	-	8
CROMO*	USA	CSW1.3	CR CSW1.3	10.1	16.9	0.283	172	1.97E-02	1.55	5.30	-2.20	-	8
CROMO*	USA	CSW1.1	CR CSW1.1	12.2	17.2	0.289	253	4.13E-06	70.79	44.55	36.31	-	8
SEO*	Costa Rica	Spring 9	SEO SP9	11.54	26.4	53.1	254.3	6.33E-05	8.57	-22.88	-21.72	-	9
Lost City	Mid-Atlantic Ridge	3871-GT15 (Vent 8)	LC 8	9.4	43	4100	0.8	4.99E-05	9	-65.09	-52.68	-60.29	10,11,12
Lost City	Mid-Atlantic Ridge	3871-GT9 (Vent C)	LC C	9.4	62	7900	0.1	4.91E-05	5.85	-62.42	-36.58	-44.64	10,11,12
Lost City	Mid-Atlantic Ridge	3865-GT7 (Vent IF)	LC IF	9.4	55	3350	0.1	5.36E-05	7.5	-53.28	-31.41	-39.31	10,11,12
Lost City	Mid-Atlantic Ridge	3876-GT9 (Vent BH)	LC BH	9.4	90	9220	0.1	3.51E-05	8.1	-63.08	-17.83	-26.57	10,11,12
Lost City	Mid-Atlantic Ridge	3876-GT7 (Vent BH)	LC BH	9.4	90	8970	0.1	3.51E-05	8.1	-62.80	-17.50	-26.24	10,11,12
Lost City	Mid-Atlantic Ridge	3879-GT7 (Vent H)	LC H	9.4	60	1270	0.6	3.02E-04	13	-51.19	-25.44	-33.45	10,11,12
Lost City	Mid-Atlantic Ridge	3864-GT10 (Vent IF)	LC IF	9.4	55	3690	1	5.36E-04	7.5	-65.66	-45.03	-52.93	10,11,12
Lost City	Mid-Atlantic Ridge	3879-GT10 (Vent H)	LC H	9.4	60	2210	2	1.01E-03	13	-62.66	-38.24	-46.26	10,11,12
Lost City	Mid-Atlantic Ridge	3866-GT11 (Vent 7)	LC 7	9.4	28	5540	4	3.03E-03	8.57	-76.05	-74.09	-81.34	10,11,12
Lost City	Mid-Atlantic Ridge	3866-GT14 (Vent 7)	LC 7	9.4	28	5430	4	3.03E-03	8.57	-75.85	-73.89	-81.14	10,11,12
Lost City	Mid-Atlantic Ridge	3871-GT10 (Vent 6)	LC 6	9.4	62	14190	9	4.42E-03	8.57	-89.59	-67.12	-75.18	10,11,12
Lost City	Mid-Atlantic Ridge	3862-GT7 (Vent 3)	LC 3	9.4	61	11580	10	4.97E-03	1.3	-92.77	-71.29	-79.33	10,11,12
Lost City	Mid-Atlantic Ridge	3878-GT14 (Vent 3)	LC 3	9.4	73	11940	26	1.12E-02	1.3	-97.80	-69.98	-78.31	10,11,12

*CROMO, Coast Range Ophiolite Microbiological Observatory; SEO, Santa Elena Ophiolite.

[†]For The Cedars spring samples, as no H_2 concentration has been reported, so the highest on-land serpentinite-hosted system H_2 concentration was used (664 μM H_2 from Hakuba Happo #1).

[‡]For the samples with TIC concentrations below the detection limit, the value of the detection limit was used (0.1 μM TIC); CO_2 concentration was calculated from TIC and pH values.

[§]For samples with no reported acetate concentrations, the average of reported concentrations was used (8.57 μM acetate).

[¶]ΔG_r, ΔG at 297 K; ΔG_{temp}, ΔG at *in situ* temperature; $\Delta G_{temp+prs}$, ΔG at *in situ* temperature and pressure (only for Hakuba [140 atm] and Lost City [88.2 atm] samples).

References:

1. Suda, K. et al. Origin of methane in serpentinite-hosted hydrothermal systems: The CH_4–H_2–H_2O hydrogen isotope systematics of the Hakuba Happo hot spring. Earth and Planetary Science Letters, 386, 112-125, (2014). https://doi.org/10.1016/j.epsl.2013.11.001
2. Suda, K. et al. Compound–and position–specific carbon isotopic signatures of abiogenic hydrocarbons from on–land serpentinite–hosted Hakuba Happo hot spring in Japan. Geochimica et Cosmochimica Acta, 206, 201-215, (2017). https://doi.org/10.1016/j.gca.2017.03.008
3. Suda, K. Origins of Hydrocarbons in On-land Serpentinization Fields and Insights into Hadean Hydrothermal Systems: Systematic Study using Stable Isotopes Ph.D. thesis, Tokyo Institute of Technology, (2016).
4. Suzuki, S. et al. Microbial diversity in The Cedars, an ultrabasic, ultrareducing, and low salinity serpentinizing ecosystem. Proceedings of the National Academy of Sciences, 110, 15336-15341, (2013). https://doi.org/10.1073/pnas.1302426110
5. Brazelton W.J. et al. Metagenomic identification of active methanogens and methanotrophs in serpentinite springs of the Voltri Massif, Italy. PeerJ., 5, e2945, (2017). https://doi.org/10.7717/peerj.2945
6. Cipolli et al. Geochemistry of high-pH waters from serpentinites of the Gruppo di Voltri (Genova, Italy) and reaction path modeling of CO_2 sequestration in serpentinite aquifers. Applied Geochemistry, 19, 787-802, (2004). https://doi.org/10.1016/j.apgeochem.2003.10.007
7. Szponar, N. et al. Geochemistry of a continental site of serpentinization, the Tablelands Ophiolite, Gros Morne National Park: a Mars analogue. Icarus, 224, 286-296, (2013). https://doi.org/10.1016/j.icarus.2012.07.004
8. Twing, K.I. et al. Serpentinization-influenced groundwater harbors extremely low diversity microbial communities adapted to high pH. Frontiers in microbiology, 8, 308, (2017). https://doi.org/10.3389/fmicb.2017.00308
9. Crespo-Medina, M. et al. Methane dynamics in a tropical serpentinizing environment: the Santa Elena Ophiolite, Costa Rica. Frontiers in Microbiology, 8, 916, (2017). https://doi.org/10.3389/fmicb.2017.00916
10. Kelly, D.S. et al. An off-axis hydrothermal vent field near the Mid-Atlantic Ridge at 30° N. Nature, 412, 145-149, (2001). https://doi.org/10.1038/35084000
11. Proskurowski, G. et al. Abiogenic hydrocarbon production at Lost City hydrothermal field. Science 319, 604–607, (2008). https://doi.org/10.1126/science.1151194
12. Lang S.Q. et al. Elevated concentrations of formate, acetate and dissolved organic carbon found at the Lost City hydrothermal field. Geochimica et Cosmochimica Acta, 74, 941-952, (2010). https://doi.org/10.1016/j.gca.2009.10.045

Table S3. Phylogeny and quality of bins from Hakuba Happo hot springs and The Cedars springs (GPS1 and BS5). Phylogeny was defined by GTDBtk (g) or comparing GTDBtk-defined phylogeny with EMBL (e) or SILVA (s). GTDBtk annotations with RED values less than 0.5 were not considered and phylogeny was checked by constructing a concatenated ribosomal protein tree (see Supplement). * Low quality bins that were only used for comparative purposes (e.g., whether a function found in a high-quality bin from HKB1 is present in HKB2 with >99% similarity)

Information				Phylogeny				Genome statistics			
Habitat	Sample	Bin	Prefix	Phylogeny (phylum)	Phylogeny (lowest level)	based on GTDBtk	GTDBtk RED value	Genome size (Mb)	Contigs (#)	Completeness (%)	Contamination (%)
Hakuba	HKB702	HKB206	DDT18	*Actinobacteria*	UBA1414 (g)	d__Bacteria;p__Actinobacteriota;c__UBA1414;o__;f__;g__;s__	0.502308	1.97	346	85.5	5.5
Hakuba	HKB701	HKB109	DDT19	*Firmicutes*	*Syntrophomonadaceae* (e)	d__Bacteria;p__Firmicutes_D;c__Dethiobacteria;o__Dethiobacterales;f__Dethiobacteraceae;	0.850189	2.98	562	73.2	6.9
Hakuba	HKB702	HKB212	DDT20	*Firmicutes*	SRB2 (s)	d__Bacteria;p__Firmicutes_D;c__UBA994;o__UBA994;f__UBA994;g__UBA994;s__	0.937622	1.75	209	89.4	0
Hakuba	HKB702	HKB214	DDT21	*Firmicutes*	*Syntrophomonadaceae* (e)	d__Bacteria;p__Firmicutes_D;c__Dethiobacteria;o__Dethiobacterales;f__;g__;s__	0.686205	2.67	112	89.4	1.1
Hakuba	HKB702	HKB210	DDT22	*Ca.* Lithacetigenota (novel)	-	*d__Bacteria;p__Coprothermobacterota;c__Coprothermobacteria;o__;f__;g__;s__*	0.417028	1.30	223	88.8	5.2
Hakuba	HKB701	HKB111	DDT23	*Ca.* Lithacetigenota (novel)	-	*d__Bacteria;p__Coprothermobacterota;c__Coprothermobacteria;o__;f__;g__;s__*	0.421002	1.24	204	83.6	0
The Cedars	GPS1 2011	GPS105*	DDT24	*Chloroflexi*	*Dehalococcoidia* (s)	d__Bacteria;p__Chloroflexota;c__Dehalococcoidia;o__SZUA-161;f__;g__;s__	0.632993	0.88	233	62	0
The Cedars	BS5 2011	BS517*	DDT25	*Chloroflexi*	*Dehalococcoidia* (s)	d__Bacteria;p__Chloroflexota;c__Dehalococcoidia;o__SZUA-161;f__;g__;s__	0.629093	1.31	304	59.7	0
The Cedars	BS5 2012	BS5B11	DDT26	*Chloroflexi*	*Dehalococcoidia* (s)	d__Bacteria;p__Chloroflexota;c__Dehalococcoidia;o__SZUA-161;f__;g__;s__	0.628746	2.51	486	85.1	3.5
The Cedars	BS5 2011	BS503	DDT27	*Chloroflexi*	*Dehalococcoidia* (s)	d__Bacteria;p__Chloroflexota;c__Dehalococcoidia;o__SZUA-161;f__;g__;s__	0.626397	1.52	342	74.1	1
The Cedars	GPS1 2012	GPS1B04*	DDT28	*Chloroflexi*	*Dehalococcoidia* (s)	d__Bacteria;p__Chloroflexota;c__Dehalococcoidia;o__SZUA-161;f__;g__;s__	0.634978	1.13	298	59.1	2.1
The Cedars	GPS1 2011	GPS109*	DDT29	*Firmicutes*	*Syntrophomonadaceae* (e)	d__Bacteria;p__Firmicutes_D;c__Dethiobacteria;o__Dethiobacterales;f__Dethiobacteraceae;	0.8487	2.70	688	85.2	9.6
The Cedars	GPS1 2012	GPS1B09	DDT30	*Firmicutes*	*Syntrophomonadaceae* (e)	d__Bacteria;p__Firmicutes_D;c__Dethiobacteria;o__Dethiobacterales;f__Dethiobacteraceae;	0.847308	2.32	503	86.9	3.9
The Cedars	BS5 2012	BS5B29	DDT34	*Firmicutes*	SRB2 (s)	d__Bacteria;p__Firmicutes_D;c__UBA994;o__UBA994;f__UBA994;g__;s__	0.828123	2.60	521	91.5	4.2
The Cedars	GPS1 2011	GPS123	DDT35	*Firmicutes*	SRB2 (s)	d__Bacteria;p__Firmicutes_D;c__UBA994;o__UBA994;f__UBA994;g__;s__	0.824263	1.51	279	92.2	3.6
The Cedars	BS5 2011	BS524	DDT36	*Firmicutes*	SRB2 (s)	d__Bacteria;p__Firmicutes_D;c__UBA994;o__UBA994;f__UBA994;g__;s__	0.820371	1.74	227	88.1	1.7
The Cedars	GPS1 2011	GPS119	DDT37	*Firmicutes*	SRB2 (s)	d__Bacteria;p__Firmicutes_D;c__UBA994;o__UBA994;f__UBA994;g__UBA994;s__	0.937791	1.95	283	89.41	1.7
The Cedars	BS5 2011	BS530	DDT38	*Firmicutes*	SRB2 (s)	d__Bacteria;p__Firmicutes_D;c__UBA994;o__UBA994;f__UBA994;g__UBA994;s__	0.941416	1.68	384	86.4	2.9
The Cedars	BS5 2012	BS5B34	DDT39	*Firmicutes*	SRB2 (s)	d__Bacteria;p__Firmicutes_D;c__UBA994;o__UBA994;f__UBA994;g__UBA994;s__	0.934183	1.69	329	88.5	2
The Cedars	BS5 2011	BS529*	DDT31	NPL-UPA2	-	*d__Bacteria;p__Ratteibacteria;c__UBA8468;o__;f__;g__;s__*	0.46067	2.02	481	74.4	7.5
The Cedars	GPS1 2012	GPS1B11	DDT32	NPL-UPA2	-	*d__Bacteria;p__;c__;o__;f__;g__;s__*	*0.381487*	2.47	583	87	8.1
The Cedars	GPS1 2011	GPS112	DDT33	NPL-UPA2	-	*d__Bacteria;p__;c__;o__;f__;g__;s__*	*0.382715*	1.81	401	85.3	3.6
The Cedars	GPS1 2012	GPS1B18*	DDT40	*Ca.* Lithacetigenota (novel)	-	*d__Bacteria;p__Coprothermobacterota;c__Coprothermobacteria;o__;f__;g__;s__*	*0.417496*	2.06	596	79.31	9.5
The Cedars	BS5 2012	BS5B28	DDT41	*Ca.* Lithacetigenota (novel)	-	*d__Bacteria;p__Coprothermobacterota;c__Coprothermobacteria;o__;f__;g__;s__*	*0.42171*	1.75	278	94.8	2.9
The Cedars	BS5 2011	BS525	DDT42	*Ca.* Lithacetigenota (novel)	-	*d__Bacteria;p__Coprothermobacterota;c__Coprothermobacteria;o__;f__;g__;s__*	*0.417261*	2.12	427	89.7	4.4

Table S4. Distribution of acetogenesis-related pathways among bins recovered from Hakuba Happo hot springs and The Cedars springs. Low quality bins are grayed out. * Missing one non-substrate-binding subunit of a multi-subunit protein complex.

Bin	Prefix	Phylum	Specific	Hydrogenase					Formate dehydrogenase					THF pathway					CO deh.	Bacterial CODH/ACS					Archaeal CODH/ACS					Hybrid CODH/ACS				Glycine reductase		Electron transport									
				hoxEFUHY	hoxEFUHYP	hydABC	hydABCD	hndABCD	fdhA-hylBC	fdhA-hylABC	fdhA	fdpAB	fdnGH-hybAB	fhs	folD	fchA	metV	metF	CooS	acsA	acsB	acsC	acsD	acsE	cdhA	cdhB	cdhC	cdhD	cdhE	acsABCDE	cdhABCD-acsCE	cdhABC-acsCDE	cdhBC-acsACDE	grdABCDE	trxAB	rnfA	rnfB	rnfC	rnfD	rnfE	rnfG	nfnAB	hdrA	hdrB	hdrC
**GPS109**	DDT29	*Firmicutes*	*Syntrophomonadaceae (e)*				+				+			+	+		+	+		+	+	+	+	+	+	+	+	+		+	+	+				+	+	+	+	+	+				
**GPS1B09**	DDT30	*Firmicutes*	*Syntrophomonadaceae (e)*			+	+							+	+		+	+	+	+		+	+	+	+	+	+					+				+	+	+	+	+	+				
**HKB109**	DDT19	*Firmicutes*	*Syntrophomonadaceae (e)*			+								+	+		+	+		+	+	+	+	+	+	+	+	+		+	+	+				+	+	+	+	+	+	+	+		
**HKB214**	DDT21	*Firmicutes*	*Syntrophomonadaceae (e)*			+	+			+		+		+	+		+	+	+	+	+	+	+	+	+	+	+	+		+	+	+				+	+	+	+	+	+		+	+	+
**GPS1B11**	DDT32	NPL-UPA2	-			+			+					+	+		+	+	+	+	+	+	+	+	+	+	+	+		+	+	+				+	+	+	+	+	+		+		
**GPS112**	DDT33	NPL-UPA2	-											+	+		+	+				+	+	+	+	+	+									+	+	+	+	+	+		+		
**BS529**	DDT31	NPL-UPA2	-			+									+		+	+				+		+												+		+	+	+	+		+	+	
**HKB206**	DDT18	*Actinobacteria*	UBA1414 (g)		+				+					+	+		+	+	+	+		+	+	+		+	+						+			+	+		+	+	+	+	+	+	+
**BS525**	DDT42	*Ca.* Lithacetigenota (novel)	-		+									+	+		+	+				+	+	+	+	+	+					+		+	+	+	+	+	+	+	+		+	+	
**BS5B28**	DDT41	*Ca.* Lithacetigenota (novel)	-		+									+	+		+	+				+	+	+	+	+	+					+		+	+	+	+	+	+	+	+		+	+	
**GPS1B18**	DDT40	*Ca.* Lithacetigenota (novel)	-		+									+	+		+					+	+	+	+	+	+					+		+	+	+	+	+		+	+		+	+	
**GPS105**	DDT24	*Chloroflexi*	*Dehalococcoidia (s)*			+				+							+	+		+			+	+	+	+		+								+		+				+	+		
**GPS1B04**	DDT28	*Chloroflexi*	*Dehalococcoidia (s)*	+		+					+					+	+	+	+															+*	+	+		+			+		+		
**BS503**	DDT27	*Chloroflexi*	*Dehalococcoidia (s)*			+					+			+	+			+		+			+	+				+								+	+	+	+		+		+	+	
**BS517**	DDT25	*Chloroflexi*	*Dehalococcoidia (s)*						+					+			+	+	+																			+				+	+		
**BS5B11**	DDT26	*Chloroflexi*	*Dehalococcoidia (s)*			+				+				+	+	+	+	+				+	+	+		+		+						+*	+	+	+	+	+		+		+	+	
**BS530**	DDT38	*Firmicutes*	SRB2 (s)			+	+		+	+				+		+																				+	+		+	+	+		+		
**GPS119**	DDT37	*Firmicutes*	SRB2 (s)			+	+							+		+																		+	+	+	+	+	+	+	+				
**HKB212**	DDT20	*Firmicutes*	SRB2 (s)			+	+							+	+	+																		+	+	+	+	+	+	+	+				
**BS5B34**	DDT39	*Firmicutes*	SRB2 (s)			+								+		+																		+	+		+		+						
**BS5B29**	DDT34	*Firmicutes*	SRB2 (s)			+	+		+	+				+	+	+																		+	+	+		+	+	+	+		+		
**GPS123**	DDT35	*Firmicutes*	SRB2 (s)			+								+	+	+																				+	+		+	+	+				
**BS524**	DDT36	*Firmicutes*	SRB2 (s)			+		+						+	+	+																				+	+	+	+	+	+				
**HKB111**	DDT23	*Ca.* Lithacetigenota (novel)	-		+*																			+										+	+	+	+	+	+	+	+		+		
**HKB210**	DDT22	*Ca.* Lithacetigenota (novel)	-		+									+									+	+										+	+	+	+	+	+	+	+				

Table S5. Locus tags for acetogenesis-related genes in select Hakuba Happo hot spring and The Cedars springs bins. For hydrogenases and formate dehydrogenases, CD-SEARCH domains are shown. Hydrogenases were confirmed to have conserved residues necessary for activity using motifs/patterns identified by Vignais and Billoud Chem Rev 2007. NiFe hydrogenase: modified L1 and L2 for Group 3d NiFe hydrogenase "E......R.CG.C...H........D" "DPC.SC..H" (only retained fully conserved residues from original motif). FeFe hydrogenase: modified P1, P2, and P3 for FeFe hydrogenase "[ST][SCM]C[CS]P.[FWY]" "PC..K..[EV]" "E.M.C..GC..G" (only retained fully conserved residues and positions with low variation [≤3 residue types]). † though not all components of the putative NADPH-dependent oxidoreductase found in Lithoacetigenota DDT41 is present in Lithoacetigenota genome DDT22, one gene with high homology (84.15% sequence identity) is detected at the edge of a contig, suggesting that the remaining two genes may be present in the DDT22 genome but was not assembled/recovered.

		Ca. Lithacetigenota		UBA1414	NPL-UPA2	*Syntrophomonadaceae*	*Syntrophomonadaceae*	*Syntrophomonadaceae*	*Dehalococcoidia*	SRB2	SRB2
		HKB210	BS5B28	HKB206	GPS1B11	HKB214	GPS1B09	HKB109	BS5B11	HKB212	GPS119
**Glycine reductase**	GrdE-TrxA	DDT22_00297	DDT41_00874	-	-	-	-	-	-	-	-
	GrdE	*DDT22_00297*	*DDT41_00874*	-	-	-	-	-	DDT26_01340	DDT20_01364	DDT37_00574
	GrdA1	DDT22_00296	DDT41_00873	-	-	-	-	-	DDT26_01344	DDT20_01365	DDT37_00573
	GrdA2	DDT22_00295	DDT41_00872	-	-	-	-	-	DDT26_01343	DDT20_01366	DDT37_00572
	GrdB	DDT22_00294	DDT41_00871	-	-	-	-	-	DDT26_01339	DDT20_01367	DDT37_00571
	GrdC	DDT22_00292	DDT41_00869	-	-	-	-	-	-	*DDT20_01369*	*DDT37_00570*
	GrdD	DDT22_00291	DDT41_00868	-	-	-	-	-	-	*DDT20_01369*	*DDT37_00570*
	GrdCD	-	-	-	-	-	-	-	-	DDT20_01369	DDT37_00570
**Thioredoxin**	TrxA	*DDT22_00297*	*DDT41_00874*	-	-	-	-	-	DDT26_00572	DDT20_01363	DDT37_00575
	TrxB	DDT22_00328	DDT41_00629	-	-	-	-	-	DDT26_00593	DDT20_01362	DDT37_00576
**Sec biosynthesis**	SelA	DDT22_00171	DDT41_00605	DDT18_01429	-	DDT21_01805	DDT30_01458	-	DDT26_01079	DDT20_01332	DDT37_00583
	SelB	DDT22_00172	DDT41_00604	DDT18_01428	-	DDT21_01806	DDT30_01459	-	DDT26_00526	DDT20_01331	DDT37_00582
**NiFe hydrogenase**	HoxY (FrhG superfamily; cl34378)	DDT22_00350	DDT41_00425	DDT18_00660	-	-	-	-	DDT26_02429	-	-
	HoxH (FrhA; COG3259)	DDT22_00351	DDT41_00426	DDT18_00659	-	-	-	-	-	-	-
	HoxU (NuoG superfamily; cl34073)	DDT22_00349	DDT41_00424	DDT18_00661	-	-	-	-	DDT26_01928	-	-
	HoxF (NuoF; COG1894 | thioredoxin/fd family; cd02980)	DDT22_00348	DDT41_00423	DDT18_00626	-	-	-	-	DDT26_00808	-	-
	HoxE (2Fe2S thioredoxin; pfam01257)	DDT22_00347	DDT41_00422	DDT18_00627	-	-	-	-	DDT26_00809	-	-
	HoxP (GltD superfamily; cl40752)	DDT22_00346	DDT41_00421	DDT18_00662	-	-	-	-	-	-	-
**FeFe hydrogenase**	HydA (NuoG; COG1034 | FeFe hydrogenase A superfamily; cl37132 | Fe hydrogenase SSU; pfam02256 | thioredoxin/fd family; cd02980)	-	-	-	-	DDT21_01978	DDT30_01055	-	DDT26_01129	DDT20_00751	-
	HydB (NuoF; COG1894 | DMSOR-beta-like superfamily; cl25360 | Thioredoxin-like superfamily; cl00388)	-	-	-	-	DDT21_01977	DDT30_01054	-	DDT26_01128	DDT20_00750	-
	HydC (2Fe2S thioredoxin; pfam01257)	-	-	-	-	DDT21_01976	DDT30_01053	-	DDT26_01127	DDT20_00752	-
	HydA (NuoG; COG1034 | FeFe hydrogenase A superfamily; cl37132 | Fe hydrogenase SSU)	-	-	-	-	-	-	-	-	DDT20_00909	DDT37_00655
	HydB (NuoF; COG1894 | DMSOR-beta-like superfamily; cl25360 | Thioredoxin-like superfamily; cl00388)	-	-	-	-	-	-	-	-	DDT20_00908	DDT37_00654
	HydC (2Fe2S thioredoxin; pfam01257)	-	-	-	-	-	-	-	-	DDT20_00907	DDT37_00653
	HydD (thioredoxin/fd family; cd02980)	-	-	-	-	-	-	-	-	DDT20_00906	DDT37_00652
	HndA (2Fe2S thioredoxin; pfam01257)	-	-	-	-	DDT21_01026	DDT30_00515	-	-	-	-
	CckA (HATPase_c; smart00387)					DDT21_01027	DDT30_00514	-	-		
	HndB (thioredoxin/fd family; cd02980)	-	-	-	-	DDT21_01028	DDT30_00513	-	-	-	-
	HndC (NuoF; COG1894 | DMSOR-beta-like superfamily; cl25360 | Thioredoxin-like superfamily; cl00388)	-	-	-	-	DDT21_01029	DDT30_00512	-	-	-	-
	HndD (NuoG; COG1034 | FeFe hydrogenase A superfamily; cl37132 | Fd hydrogenase SSU; pfam02256)	-	-	-	-	DDT21_01030	DDT30_00511	-	-	-	-
	HndA (2Fe2S thioredoxin; pfam01257)	-	-	-	-			DDT19_00831	-	-	-
	CckA (HATPase_c; smart00387)							DDT19_00832	-		
	HndB (thioredoxin/fd family; cd02980)	-	-	-	-			DDT19_00833	-	-	-
	HoxP (GltD superfamily; cl40752)	-	-	-	-			DDT19_00834	-	-	-
	HndD (NuoG; COG1034 | FeFe hydrogenase A superfamily; cl37132 | Fd hydrogenase SSU; pfam02256)	-	-	-	-			DDT19_00835	-	-	-
**Formate dehdyrogenase**	FdhA (Formate dehydrogenase A; TIGR01591)	-	-	DDT18_01054	DDT32_00315	DDT21_02578	-	-	DDT26_01503	-	-
	(FdhA2 [YjgC; COG3383])	-	-	-	-	DDT21_02579	-	-	DDT26_01504	-	-
	HydB-like (NuoF; COG1894 | DMSOR-beta-like superfamily; cl25360 | Thioredoxin-like superfamily; cl00388)	-	-	DDT18_01056	DDT32_00311	DDT21_02575	-	-	DDT26_01501	-	-
	HydC-like (2Fe2S thioredoxin; pfam01257)	-	-	DDT18_01055	DDT32_00312	DDT21_02576	-	-	DDT26_01502	-	-
	MetV	-	-	DDT18_01057	DDT32_00310	-	-	-	-	-	-
	MetF	-	-	-	DDT32_00309	-	-	-	-	-	-
	FdhA (Formate dehydrogenase A; TIGR01591)	-	-	-	-	DDT21_01601	-	-	-	-	-
	HydC-like (2Fe2S thioredoxin; pfam01257)	-	-	-	-	DDT21_01600	-	-	-	-	-
	Putative NADP-binding formate dehydrogenase subunit beta FdpB (thioredoxin/fd family; cd02980 | NuoF; COG1894 | GltD superfamily; PRK12771)	-	-	-	-	DDT21_01599	-	-	-	-	-
	FdhA (Molybdopterin-binding syperfamily; COG3383)	-	-	-	-	DDT21_01601	-	-	-	-	-
	Putative NADP-binding formate dehydrogenase subunit beta FdpB (thioredoxin/fd family; cd02980 | NuoF; COG1894 | GltD superfamily; PRK12771)	-	-	-	-	DDT21_01599	-	-	-	-	-
	HydC (2Fe2S thioredoxin; pfam01257)	-	-	-	-	DDT21_01164	-	-	-	-	-
	HydB (NuoF; COG1894 | DMSOR-beta-like superfamily; cl25360 | Thioredoxin-like superfamily; cl00388)	-	-	-	-	DDT21_01163	-	-	-	-	-
	Putative NADP-binding formate dehydrogenase alpha and beta fusion protein FdpAB (GltD; PRK12814 | Molybdopterin-binding superfamily; COG3383)	-	-	-	-	DDT21_01162	-	-	-	-	-
**C1 metabolism**	FchA	-	-	-	-	-	-	-	DDT26_02662	DDT20_01400	DDT37_00119
	Fhs	DDT22_01344	DDT41_01181	DDT18_01669	DDT32_00091	DDT21_00652	DDT30_01889	DDT19_01668	DDT26_02308	DDT20_01401	DDT37_00120
	FolD	-	DDT41_01485	DDT18_01668	DDT32_00071	DDT21_01161	DDT30_01019	DDT19_01973	DDT26_02307	DDT20_00767	-
	MetV	-	DDT41_01411	DDT18_00798	DDT32_00310	DDT21_00074	DDT30_00161	DDT19_00973	DDT26_01099	-	-
	MetF	-	DDT41_01410	DDT18_00797	DDT32_00309	DDT21_00075	DDT30_00160	DDT19_00974	DDT26_01100	-	-
	MtmB	-	-	DDT18_01085	-	-	-	-	-	-	-
	MtmC	-	-	DDT18_01084	-	-	-	-	-	-	-
**Acetyl-CoA synthase / CO dehydrogenase**	CooS	-	-	DDT18_01860(?)	DDT32_02123	DDT21_01084	DDT30_01952	-	-	-	-
	AcsA	-	-	-	DDT32_00844	DDT21_00067	DDT30_01473	DDT19_01000	-	-	-
	AcsB	-	-	-	DDT32_00845	DDT21_00068	-	DDT19_02607	-	-	-
	AcsC	-	-	-	DDT32_00795	DDT21_00069	DDT30_00165	DDT19_00968	-	-	-
	AcsD	-	-	-	DDT32_00843	DDT21_00071	DDT30_00164	DDT19_00970	DDT26_02597	-	-
	AcsE	-	-	-	DDT32_00794	DDT21_00072	DDT30_00163	DDT19_00971	DDT26_02596	-	-
	CdhA	-	DDT41_00748	-	DDT32_00095	DDT21_00063	DDT30_00615	DDT19_02581	-	-	-
	CdhB	-	DDT41_00750	-	DDT32_00094	DDT21_00064	DDT30_00616	DDT19_02580	-	-	-
	CdhC	-	DDT41_00751	-	DDT32_00093	DDT21_00065	DDT30_00617	-	-	-	-
	AcsC	-	DDT41_00752	-	DDT32_00092	DDT21_00066	DDT30_00618	-	-	-	-
	AcsD	-	DDT41_01017	-	DDT32_00096	DDT21_00062	DDT30_00614	-	-	-	-
	AcsE	-	DDT41_01016	-	DDT32_00098	DDT21_00061	DDT30_00613	-	-	-	-
	AcsA	-	-	DDT18_00628	-	-	-	-	-	-	-
	CdhB	-	-	DDT18_01643	-	-	-	-	-	-	-
	CdhC	-	-	DDT18_01644	-	-	-	-	-	-	-
	AcsC1	-	-	DDT18_01645	-	-	-	-	-	-	-
	AcsC2	-	-	DDT18_01462	-	-	-	-	-	-	-
	AcsD1	-	-	DDT18_01460	-	-	-	-	-	-	-
	AcsD2	-	-	DDT18_01461	-	-	-	-	-	-	-
	AcsE	-	-	DDT18_00130	-	-	-	-	-	-	-
**Substrate-level phosphorylation**	Pta	DDT23_18: 12235-12716	DDT41_00043	DDT18_01737	-	-	-	-	-	DDT20_01393	DDT37_00439
	Ack	DDT22_00702	DDT41_00042	-	-	-	-	-	-	DDT20_01392	DDT37_00438
	Ack	-	DDT41_00044	-	-	-	-	-	-	DDT20_01394	DDT37_00441
	Acs1	-	DDT41_01004	DDT18_01738*	DDT32_00571	DDT21_01032	DDT30_00052	-	DDT26_01691	-	-
	Acs2	-	-	-	DDT32_00005	DDT21_01574	-	-	DDT26_01010	-	-
	ActP	-	-	DDT18_01988	-	DDT21_00671	-	-	-	-	-
	(split)	-	-	DDT18_01987	-	-	-	-	-	-	-
**Antiporters**	MrpB	-	-	-	-	DDT21_00144	DDT30_00118	DDT19_01170	DDT26_01065	DDT20_00034	DDT37_00374
	MrpC	-	-	-	-	DDT21_00145	DDT30_00117	DDT19_01171	DDT26_01066	DDT20_00033	DDT37_00373
	MrpD	-	-	-	-	DDT21_00146	DDT30_00116	DDT19_01172	DDT26_01067	DDT20_00032	DDT37_00372
	MrpE	-	-	-	-	DDT21_00147	DDT30_00115	DDT19_01173	DDT26_01068	DDT20_00031	DDT37_00371
	MrpF	-	-	-	-	DDT21_00148	DDT30_00114	DDT19_01174	DDT26_01069	DDT20_00030	DDT37_00370
	MrpG	-	-	-	-	DDT21_00149	DDT30_00113	DDT19_01175	DDT26_01070	DDT20_00029	DDT37_00369
	MrpA/D	-	-	-	-	DDT21_00150	DDT30_00112	DDT19_01176	DDT26_01071	DDT20_00028	DDT37_00368
	MrpA/D	-	-	-	-	DDT21_00151	DDT30_00111	DDT19_01177	-	-	-
	MrpA/D	-	-	-	-	DDT21_00152	DDT30_00110	DDT19_01178	-	-	-
	MnhA	DDT22_00283	DDT41_00383	-	-	DDT21_00151	-	-	-	-	-
	MnhA?	DDT22_00284	-	-	-	-	-	-	-	-	-
	MnhB	DDT22_00285	DDT41_00386	-	-	DDT21_00148	-	DDT19_01174	-	-	-
	MnhC	DDT22_00286	DDT41_00385	-	-	DDT21_00149	-	DDT19_01175	-	-	-
	MnhD	DDT22_00287	DDT41_00384	-	-	DDT21_00150	-	DDT19_01176	-	-	-
	MnhE	DDT22_00288	DDT41_00391	-	-	DDT21_00143	-	DDT19_01169	-	-	-
	MnhF?	DDT22_00289	DDT41_00390	-	-	DDT21_00144	-	DDT19_01170	-	-	-
	MnhG	DDT22_00290	DDT41_00389	-	-	DDT21_00145	-	DDT19_01171	-	-	-
	NhaD	-	-	-	DDT32_01888	-	-	-	-	-	-
	KefB	-	-	-	-	DDT21_00752	-	-	-	-	-
	KefC	-	-	-	-	DDT21_00753	-	-	-	-	-
	KhtT	-	-	-	-	DDT21_00754	-	-	-	-	-
**Electron transport**	RnfC	DDT22_00128	DDT41_00161	-	DDT32_00181	DDT21_00136	-	DDT19_01783	DDT26_00730	DDT20_00529	DDT37_00641
	RnfD	DDT22_00127	DDT41_00160	DDT18_01397	DDT32_00180	DDT21_00137	DDT30_00125	DDT19_01782	DDT26_01058	DDT20_00528	DDT37_00640
	RnfG	DDT22_00126	DDT41_00159	DDT18_01398	DDT32_00179	DDT21_00138	DDT30_00124	DDT19_01781	DDT26_01059	DDT20_00037	DDT37_00376
	RnfE	DDT22_00125	DDT41_00158	DDT18_01399	DDT32_00178	DDT21_00139	DDT30_00123	DDT19_01780	-	DDT20_00527	DDT37_00639
	RnfA	DDT22_00124	DDT41_00157	DDT18_01400	DDT32_00177	DDT21_00140	DDT30_00122	DDT19_01779	DDT26_01062	DDT20_00526	DDT37_00638
	RnfB	DDT22_00123	DDT41_00156	DDT18_01401	DDT32_00564	DDT21_00141	-	DDT19_01778	DDT26_01063	DDT20_00525	DDT37_00637
	HydA	-	-	-	-	-	-	DDT19_01777	-	-	-
	NfnA	-	-	DDT18_01230	-	-	-	DDT19_00912	-	-	-
	NfnB	-	-	DDT18_01231	-	-	-	DDT19_00913	-	-	-
	HydC-like (2Fe2S thioredoxin; pfam01257)	†	DDT41_00298	-	DDT32_00103	-	-	-	DDT26_00809	-	-
	HydB-like (thioredoxin/fd family; cd02980 | NuoF; COG1894 | DMSOR-beta-like; cl25360)	†	DDT41_00299	-	DDT32_00102	-	-	-	DDT26_00808	-	-
	Putative NADPH-binding redox protein (GltD; cl31397)	DDT22_00846 (partial)	DDT41_00300	-	DDT32_00101	-	-	-	DDT26_00807	-	-
**Other Energy conservation**	Bcd	-	-	DDT18_01220	-	-	-	-	-	DDT20_00891	DDT37_00293
	EtfB	-	-	DDT18_01219	-	-	-	-	-	DDT20_00890	DDT37_00292
	EtfA	-	-	DDT18_01218	-	-	-	-	-	DDT20_00889	DDT37_00291
**ATP synthase (A-type)**	subunit A	-	DDT41_01328	DDT18_00494	DDT32_00513	DDT21_01718	DDT30_00226	DDT19_00643	DDT26_00842	DDT20_00644	DDT37_00801
	subunit B	-	DDT41_01329	DDT18_00493	DDT32_00514	DDT21_01717	DDT30_00225	DDT19_00644	DDT26_00840	DDT20_00643	DDT37_00802
	subunit C	DDT22_00643	-	DDT18_00496	DDT32_00511	DDT21_01720	DDT30_00228	DDT19_00641	-	DDT20_00646	DDT37_00799
	subunit D	-	DDT41_01330	DDT18_00492	DDT32_00515	DDT21_01716	DDT30_00224	DDT19_00645	-	DDT20_00642	DDT37_00803
	subunit E	DDT22_00644	DDT41_00678	DDT18_00497	DDT32_00510	DDT21_01721	DDT30_00227	DDT19_00640	-	DDT20_00647	DDT37_00798
	subunit F	DDT22_00642	DDT41_01327	-	-	-	-	-	-	DDT20_00650	DDT37_00795
	subunit G	-	-	DDT18_00495	DDT32_00512	DDT21_01719	-	DDT19_00642	-	DDT20_00645	DDT37_00800
	subunit I	DDT22_00646	DDT41_00676	DDT18_00499	DDT32_00508	DDT21_01723	-	-	-	DDT20_00649	DDT37_00796
	subunit K	DDT22_00645	DDT41_00677	DDT18_00498	DDT32_00509	DDT21_01722	DDT30_00230	DDT19_00639	DDT26_00848	DDT20_00648	DDT37_00797
**ATP synthase (F-type)**	alpha	-	-	-	-	-	-	DDT19_01308	-	-	-
	beta	-	-	-	-	-	-	DDT19_01306	-	-	-
	gamma	-	-	-	-	-	-	DDT19_01307	-	-	-
	delta	-	-	-	-	-	-	DDT19_01309	-	-	-
	epsilon	-	-	-	-	-	-	DDT19_01305	-	-	-
	a	-	-	-	-	-	-	DDT19_01312	-	-	-
	b	-	-	-	-	-	-	DDT19_01310	-	-	-
	c	-	-	-	-	-	-	DDT19_01311	-	-	-

Table S6. Environmental paramers and chemical coposition of Hakuba Happo spring water (artificially pumped from a drilling well named Happo #3) used for microbiological analysis**.**

	July 2016	October 2016	October 2017
pH	10.95	10.80	10.67
Temperature (°C)	47.5	47.4	45.6
ORP (mV)	-435	-432	-453
electrical conductivity (mS/m)	47.7	43.6	51.7
dissolved oxygen (mg/L)	<dl	<dl	<dl
Na^+ (ppm)	32	30	<dl
K^+ (ppm)	<dl	<dl	<dl
Ca^{2+} (ppm)	<dl	<dl	<dl
NO_3^- (ppm)	<dl	<dl	<dl
NH_3 (µM)	-	-	2.9
Amino acids			
Aspartic Acid (nM)	-	-	<dl
Threonine (nM)	-	-	<dl
Serine (nM)	-	-	<dl
Glutamic Acid (nM)	-	-	<dl
Glycine (nM)	-	-	5.4 ± 1.6
Alanine (nM)	-	-	<dl
Cysteine (nM)	-	-	<dl
Valine (nM)	-	-	<dl
Methionine (nM)	-	-	<dl
Isoleucine (nM)	-	-	<dl
Leucine (nM)	-	-	<dl
Tyrosine (nM)	-	-	<dl
Phenylalanine (nM)	-	-	<dl
Histidine (nM)	-	-	<dl
Lysine (nM)	-	-	<dl
Arginine (nM)	-	-	<dl

<dl, below our quantification limit; -, no data.
